# Comparisons and Contrasts in a Complete Set of Alkali Metal Cumyl Structures

**DOI:** 10.1021/acs.inorgchem.6c00468

**Published:** 2026-03-27

**Authors:** Paul D. L. Ferguson, David E. Anderson, Eva Hevia, Thomas M. Horsley Downie, Alan R. Kennedy, Stuart D. Robertson, Catherine E. Weetman, Robert E. Mulvey

**Affiliations:** † Department of Pure and Applied Chemistry, 3527 University of Strathclyde, 295 Cathedral Street, Glasgow G1 1XL, U.K.; ‡ Department für Chemie und Biochemie, 27210 Universität Bern, Freiestrasse 3, 3012 Bern, Switzerland

## Abstract

Alkali-metal benzyl complexes derived from toluene are known to vary their metal–ligand coordination mode as a function of the alkali-metal, with the metal transitioning from a typical σ bond to the anionic CH_2_ for lithium toward an interaction with the delocalized pi system of the aromatic ring as the metal gets larger and softer. Here, by switching to cumene, we report the charge-localizing effect of replacing the hydrogen atoms at the formally carbanionic carbon CH_2_ with electron-donating methyl groups in C­(Me)_2_. NMR spectroscopic studies reveal competitive ring-metalation occurs, at the meta and para positions, alongside α-metalation on using an alkyl lithium base, with the meta- and α-isomers crystallographically characterized as a solvated dimer and monomer, respectively. Using Lochmann-Schlosser type base pairs to access the heavier alkali-metal complexes unveils only α-metalation. The presence of the methyl groups limits the variation in metal–ligand bonding, their electron-donating properties forcing the delocalization of the negative charge into the ring resulting in M-Ph interactions and sp^2^ hybridization at the formally deprotonated α-carbon regardless of the metal used. Considerable variation in aggregation state is observed with monomeric (Na), polymeric (K, Cs) and tetrameric (Rb) motifs identified in the solid-state.

## Graphical abstract

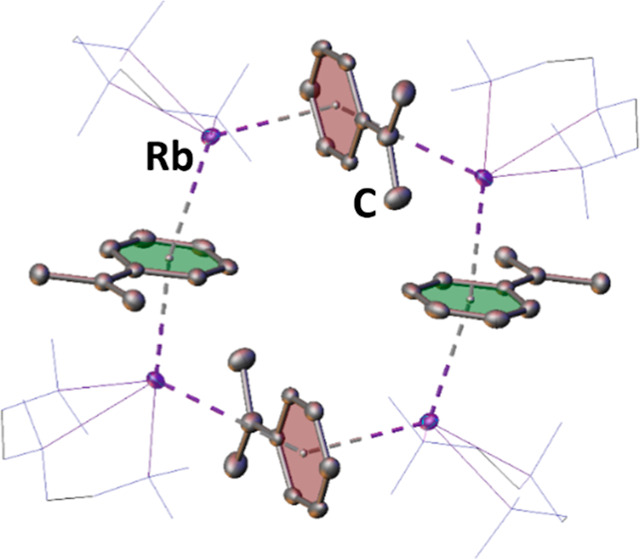

## Introduction

Though organolithium compounds have received most attention because of their phenomenal utility in synthesis over the past 100 years,^1^ the organometallic chemistry of the middleweight alkali metals sodium and potassium is attracting new interest for the purpose of advancing sustainability,^2–5^ while organoelement compounds of the heavyweights rubidium and cesium have rather surprisingly performed well in some, albeit limited, studies to date in homogeneous catalytic reactions.^6–9^ Therefore, it has become increasingly important to include the full set of nonradioactive alkali metals in studies. One way we have been carrying this out is to compare and contrast the bonding undertaken by the different metals when they interact with anionic ligands which present an opportunity for either σ- or π-ligation. This is best exemplified by the benzyl (PhCH_2_^–^) anion, generated via lateral metalation of toluene. Many of such species form supramolecular structures, exhibiting a mixture of σ- and π-interactions that propagate the infinite chain, the most pertinent example being the THF-solvate of benzylpotassium.^10^ However, we were able to exploit a bulky Lewis donor to prevent such polymerization, stripping away the secondary propagating interactions to reveal the true nature of the primary metal-anion interactions within the monomeric unit, for lithium, sodium and potassium.^11,12^ Specifically, this showed that the benzyl anion bonds to lithium via a σ-bond to the CH_2_ anion but sees migration of the alkali-metal toward the π-system of the aromatic ring as the metal gets larger and softer, with concomitant delocalization of negative charge away from the lateral CH_2_ group and into the C_6_ ring. This preference of the heavier alkali-metals to bond to softer π-systems is expected and has been coming to the fore recently in a variety of different scenarios, including for example in aromatic Cs-amides where the alkali-metal favors bonding to the aromatic ring of Dipp­(Me_3_Si)­N^–^ over the formally negatively charged nitrogen^13^ or in bimetallic low-valent aluminum complexes where such interactions stitch these reactive species into discrete dimers.^14–17^

In this new work we consider the related substituted benzyl compound made from cumene (isopropylbenzene). The metalation of cumene with alkali-metal alkyl reagents has been visited several times since the mid-20th century with contrasting results (see Table S1 for a summary of conditions, yields and observations),^18–28^ with disparities when using the same base often ascribed to different methods of base preparation. The presence of the electron-donating methyl groups on the lateral carbon atom increases its p*K*_a_, bringing it closer to those of the poorly acidic ring CH units and introducing the potential for competitive, nonselective metalations.

In all previous cases, analysis was carried out via an organic workup of the metalated intermediates followed by identification of the quenched products with no focus on the more reactive metalated intermediates formed prior to quenching. What is clear from all these previous studies is that meta and para isomeric products dominate, particularly when using a lithium base (e.g., entries 16, 17, 21–24), with alpha metalation becoming dominant upon using sodium and potassium bases. However, there is some disparity even when using sodium and potassium with ^n^amyl sodium favoring ring metalation (entries 9–11) whereas ^n^amyl potassium (entries 12–14) or phenyl sodium (entry 26) favoring lateral metalation. Furthermore, to the best of our knowledge there has been no previous work until our study here using the heaviest alkali-metals rubidium and cesium.

Consequently, considering the vast amount of data already generated via organic quenching, and given our interest in this area, we felt compelled to pursue a combined NMR and single crystal X-ray diffraction (SCXRD) study on a complete set of alkali-metal cumyl intermediate complexes. We wanted to include the heavier group 1 metals rubidium and cesium in the study as their individuality is increasingly being recognized in recent alkali-metal research,^29–34^ with growing cognisance among some researchers that the alkali-metal family can no longer be considered as all being the same - a mindset shift that we are particularly keen to encourage.

## Results and Discussion

A complete set of alkali-metal cumyl complexes were targeted by deprotonating cumene with the alkyl bases ^n^BuM (M = Li, Na) or via a Lochmann-Schlosser ^n^BuLi/MOR superbase approach (M = K, R = ^t^Bu; M = Rb, Cs, R = ^t^Amyl, Scheme 1). For lithium and sodium, the common Lewis donors TMEDA and PMDETA, respectively, were added to promote deprotonation reactions, with solvated complexes obtained in reasonable yields (37 and 52% respectively). Donor addition was necessary for the synthesis of benzylithium from toluene;^11^ that it was also necessary for our sodium reaction here reflects the lower acidity of cumene versus that of toluene. Bryce-Smith previously noted that ethyllithium does not metalate cumene even at 90 °C for 30 min.^22^ For the heavier alkali-metals K–Cs, the unsolvated metal cumyl complex precipitated from cumene and could be isolated via filtration in good yield (43–94%). Next, PMDETA was added to a small portion of the compound suspended in parent cumene to aid redissolution in attempts to grow crystals of a suitable quality to enable the determination of as yet unknown alkali metalated cumyl structures by SCXRD studies. Pleasingly, these methods were successful leading to the determination of six such crystal structures.

**Scheme 1:**
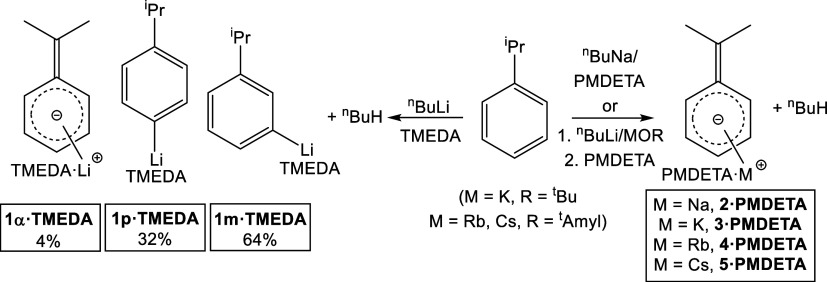
Synthesis of New Alkali-Metal Complexes^a^

a All complexes are displayed empirically rather than including aggregation state.

### Single Crystal X-ray Diffraction Study

Crystallization of lithium complex **1** yielded a mixture which contained predominantly colorless crystals mixed with some small red rods. The meta-lithiation of cumene (**1m·TMEDA**) was confirmed by SCXRD on one of the colorless crystals in the batch (Figure 1).

**Figure 1:**
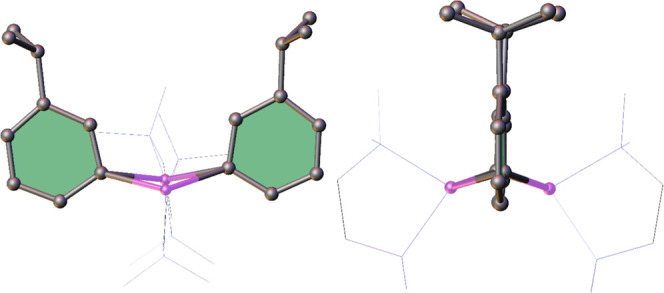
Molecular structure of dimeric **1m·TMEDA**. TMEDA molecules have been depicted as wireframes and hydrogen atoms have been omitted for clarity.

Although the data are of insufficient quality to discuss bond metrics, the connectivity is definite, comprising a dimeric arrangement with each lithium solvated by a bidentate TMEDA molecule. The four-membered [LiC]_2_ ring shows clear deviation from planarity, with the two isopropyl groups both lying on the same side of the molecule in a (noncrystallographic) cisoid C2 symmetry rather than sitting opposite one another related via a transoid inversion center.^35^ This deviation from planarity is precedented in phenyllithium structures, appearing in TMEDA solvated PhLi^36^ and *o*-EtSC_6_H_4_Li,^37^ though not in *o*-CF_3_C_6_H_4_Li.^38^ Switching to larger or higher denticity donor ligands also tends to force the four-membered ring toward planarity,^39–41^ as does using sodium in place of lithium.^28,42,43^

SCXRD studies on the red crystals established them to be the laterally metalated isomer **1α·TMEDA** (see Figure 2a for the structure and Table 1 for pertinent bond parameters). In contrast to the situation in benzyllithium,^44–50^ here the lithium cation has migrated to the π-face of the aromatic ring, intimating that the negative charge has delocalized away from the deprotonated α-carbon and into the ring, akin to the case of benzylpotassium.

**Figure 2:**
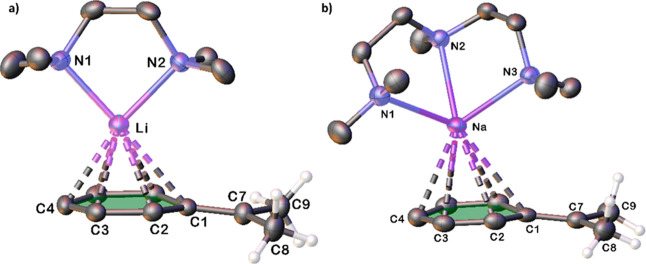
Molecular structure of (a) **1α·TMEDA** and (b) **2·PMDETA**. Ellipsoids drawn at 50% probability and selected hydrogen atoms have been omitted for clarity. Dashed lines represent AM-π-arene contacts.

**Table 1:** Selected Bond Parameters (in Å and ^o^) of Laterally-Metalated Cumyl Complexes **1**-**5**

	**1α·TMEDA**	**2·PMDETA**	**3·PMDETA**	**4·PMDETA** (Rb1)	**4·PMDETA** (Rb2)	**5·PMDETA**	cumene
M-C_ipso_	2.598(2)	3.077(1)	3.369(2)	3.601(1)	3.545(1)	3.330(7)	-
M-C_ortho_	2.417(2)	2.862(1)	3.236(2)	3.641(1)	4.387(1)	3.420(7)	-
	2.406(2)	2.865(1)	3.140(2)	3.309(1)	3.217(1)	3.596(8)	
M-C_meta_	2.341(2)	2.736(1)	3.119(2)	3.519(2)	4.835(1)	3.704(8)	-
	2.331(2)	2.750(1)	3.029(2)	3.193(1)	3.803(1)	3.879(8)	
M-C_para_	2.351(2)	2.737(1)	3.054(2)	3.326(1)	4.623(2)	3.932(10)	-
M-C_cent_	1.950(2)	2.465(1)	2.826(1)	3.131(1)	3.861(1)	3.370(3)	-
M’-C_ipso_	-	-	3.966(2)	3.593(1)	3.321(1)	3.726(7)	-
M’-C_ortho_	-	-	4.841(2)	3.610(1)	3.336(1)	3.533(7)	-
			3.134(2)	3.335(1)	3.262(1)	3.664(8)	
M’-C_meta_	-	-	4.949(2)	3.476(1)	3.326(1)	3.423(8)	-
			3.326(2)	3.223(1)	3.244(1)	3.584(8)	
M’-C_para_	-	-	4.297(2)	3.301(1)	3.292(1)	3.493(9)	-
M’-C_cent_	-	-	3.895(1)	3.122(1)	2.978(1)	3.285(3)	-
M-N	2.104(2)	2.513(1)	2.932(1)	3.043(1)	3.005(1)	3.195(7)	-
	2.081(2)	2.502(1)	2.839(1)	3.137(1)	3.142(1)	3.368(6)	
		2.463(1)	2.834(1)	3.019(1)	3.016(1)	3.218(6)	
C_ipso_-C_α_	1.374(2)	1.375(1)	1.370(2)	1.377(2)	1.379(2)	1.365(11)	1.517(3)
C_α_-C_Me_	1.500(2)	1.502(1)	1.501(3)	1.502(2)	1.508(2)	1.506(11)	1.514(3)
	1.507(2)	1.499(1)	1.506(3)	1.508(2)	1.508(2)	1.517(11)	1.523(3)
C_ipso_-C_o_	1.462(2)	1.458(1)	1.461(2)	1.457(2)	1.460(2)	1.464(10)	1.405(2)
	1.464(2)	1.459(1)	1.464(2)	1.462(2)	1.457(2)	1.457(10)	1.384(3)
C_*o*_-C_*m*_	1.374(2)	1.374(1)	1.376(3)	1.384(2)	1.371(2)	1.351(11)	1.389(3)
	1.380(2)	1.380(1)	1.370(3)	1.377(2)	1.375(2)	1.359(12)	1.384(3)
C_*m*_-C_*p*_	1.408(2)	1.407(1)	1.407(3)	1.404(2)	1.406(2)	1.405(12)	1.380(3)
	1.407(2)	1.403(1)	1.408(3)	1.400(2)	1.406(2)	1.388(12)	1.382(3)
Σ< C_α_	360.00	359.88	359.76	359.89	359.85	360.0	334.29

Crystal structures of alkali metal complexes **2**-**5** (Figures 2b and 5 and Table 1) likewise confirmed that in each case the metal is ligated to the anion through its π-system and that each metal center is solvated by a tridentate PMDETA molecule. Interestingly, this is the opposite of the trend witnessed in a phenyl-substituted benzyl-type anion derivative (that is metalating diphenylmethane, Ph_2_CH_2_) where the presence of the additional electron withdrawing phenyl ring keeps the negative charge mainly localized at C_α_.^51^ Venugopal recently reported sodium interacting with the π-system of the anion generated by lateral metalation of ethylbenzene, that is with only one electron-donating methyl group bound to the deprotonated α-carbon.^52^

Beyond these similarities in cation–anion interaction and cation solvation, these structures demonstrate considerable variety in their solid-state structures. Sodium complex **2·PMDETA** is monomeric, akin to the lithium complex (Figure 2b). Potassium complex **3·PMDETA** adopts a zigzag chain motif (Figure 3), propagating via additional interactions to the phenyl ring of the next unit. Supramolecular structures are not uncommon in benzyl alkali-metal complexes, for example being present in the OEt_2_ ^44^ or THF solvates^46^ of benzyllithium, or the PMDETA solvate of benzylsodium,^53^ or benzylpotassium^54^ but in these cases propagation is favored via M-CH_2_ interactions. The most unique structure in our set, the rubidium complex, **4·PMDETA**, crystallizes as an eyecatching cyclic tetramer. Such structures are exceptionally rare but precedented in the literature via the lighter alkali metal complexes [BnLi·Me_2_NCH_2_CH_2_OMe]_4_,^48^ [BnNa·TMEDA]_4_ ^55^ and the heterometallic [BnLi·(TMEDA)­BnNa·(TMEDA)]_2_.^56^ However, only one Rb complex, namely 4-^n^Bu-4-^t^Bu-2,6-diphenyl-1,4-dihydro-*s*-triazinido-1-rubidium is found to adopt a similar cyclo-tetrameric structure, which also exhibits M-Ph interactions in the solid state.^57^ Again, these lighter alkali-metal tetramers are stitched together via M-CH_2_ interactions whereas **4·PMDETA** prefers π-interactions to each neighboring cumyl anion as shown in Figure 4. PMDETA-solvated benzylrubidium^54^ forms an infinite supramolecular chain structure like that of **3·PMDETA**, although the Rb-benzyl interactions are η^6^ to one ring and η^3^ to the CH_2_-C_ipso_-C_ortho_ unit, suggesting greater charge delocalization into the ring in **4·PMDETA** due to the electron-releasing methyl groups on C_α_, although their greater steric profile may also contribute. Finally, cesium complex **5·PMDETA** (Figure 5) forms a supramolecular structure akin to that of the potassium complex **3·PMDETA**, albeit it with a slightly different hapticity of the M-Ph interaction (vide infra).

**Figure 3:**
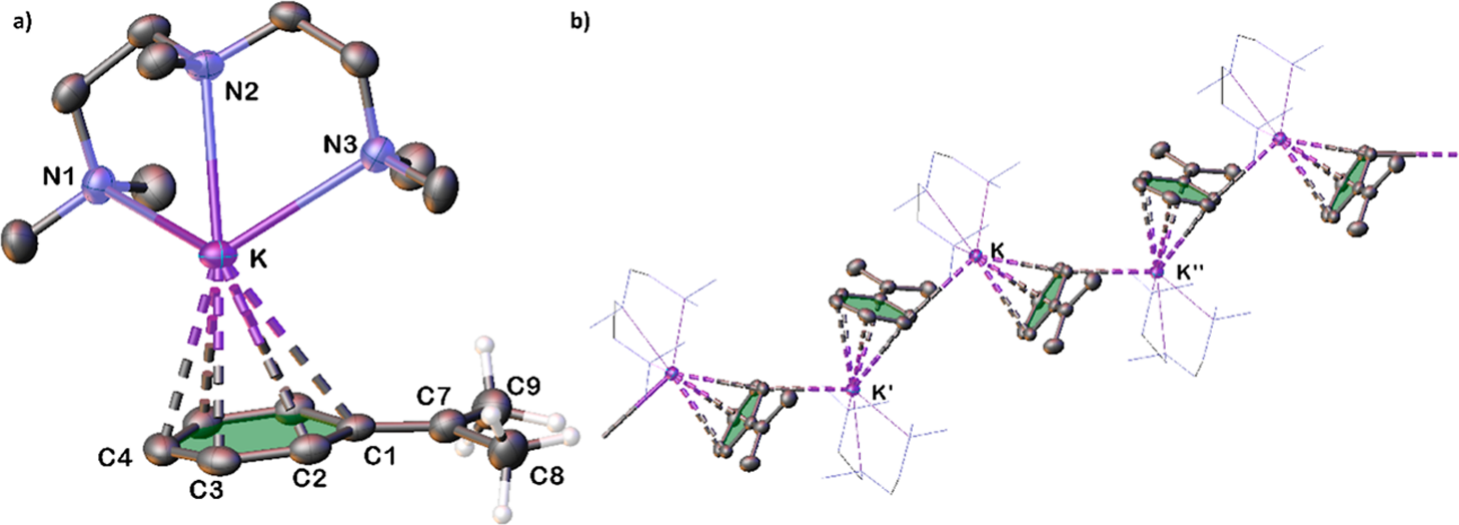
Solid-state structure of **3·PMDETA** showing (a) the asymmetric unit and (b) a section of the supramolecular structure. Thermal ellipsoids drawn at 50% probability, PMDETA molecules have been depicted as wireframes and selected hydrogen atoms have been omitted for clarity. Dashed lines represent AM-π-arene contacts.

**Figure 4:**
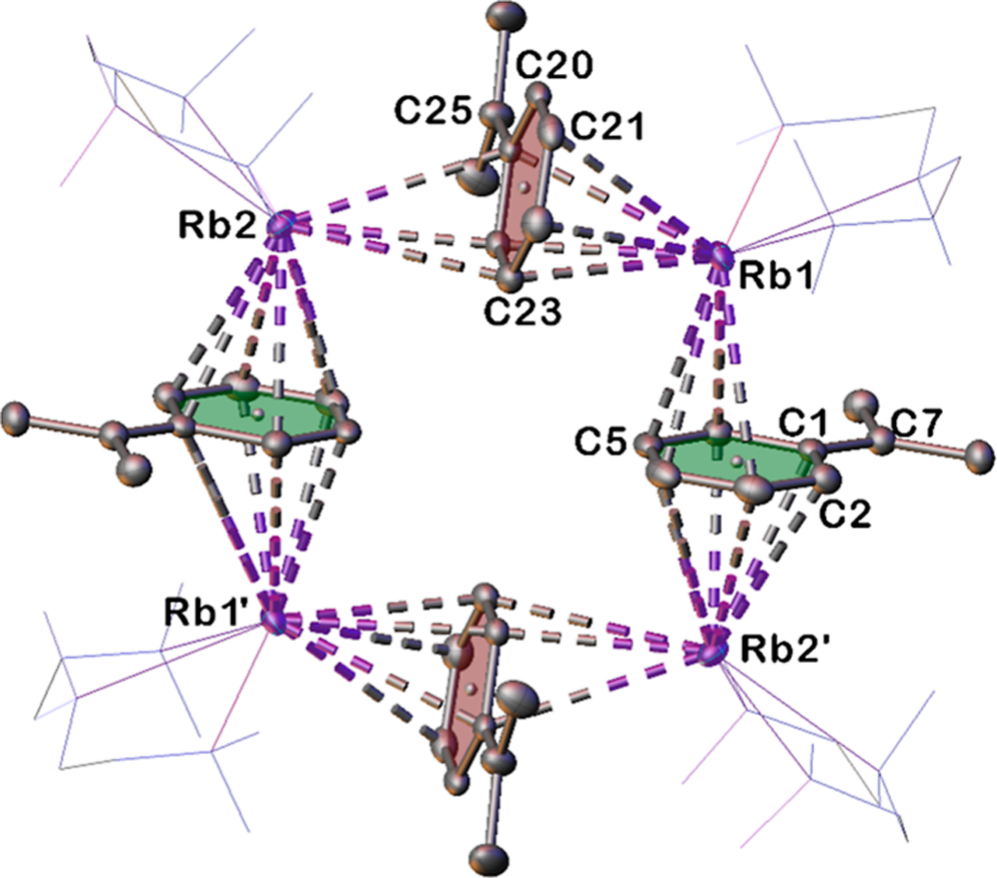
Tetrameric structure of centrosymmetric **4·PMDETA**. Ellipsoids drawn at 50% probability, PMDETA molecules have been depicted as wireframes and hydrogen atoms have been omitted for clarity. Dashed lines represent AM-π-arene contacts. Red and green rings are used to distinguish crystallographically independent cumyl ligands.

**Figure 5:**
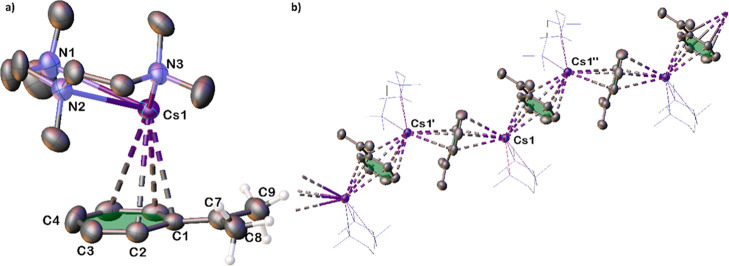
Solid-state structure of **5·PMDETA** showing (a) nondisordered section of the asymmetric unit and (b) a section of the supramolecular framework. Thermal ellipsoids drawn at 50% probability, PMDETA molecules have been depicted as wireframes and selected hydrogen atoms have been omitted for clarity. Dashed lines represent AM-π-arene contacts.

As shown in Table 1, there is little difference between the bond distances within the cumyl anions of all laterally metalated structures, regardless of metal identity or aggregation state. To identify any changes in our anions to parent cumene, we sought a structure in the Cambridge Structural Database (CSD)^58^ which contained a nondisordered molecule of cumene for comparison, the bond distances of such a molecule are also contained in Table 1 for comparison.^59^ The major changes in the cumyl anions are shortening of the C_α_-C_ipso_ bond [mean 1.373 vs 1.517(3)­Å in cumene] with concomitant lengthening of the C_ipso_-C_*o*_ bonds (1.460/1.394 Å). The C_*o*_-C_*m*_ bonds contract (1.373/1.386 Å) whereas C_*m*_-C_*p*_ bonds lengthen (1.404/1.381 Å) but these changes are less pronounced. Overall, this suggests delocalization of the negative charge across the C_*o*_-C_*m*_-C_*p*_-C_*m’*_-C_*o’*_ unit with considerable double bond character in the C_α_-C_ipso_ bond (Figure 6). The sum of the bond angles at C_α_ support the double bond character of the C_α_-C_ipso_ bond, with planarity seen in all the metal complexes (mean, 359.9 °) as opposed to a more typical tetrahedral environment (Σ< = 334.29 °) in the neutral cumene molecule.

**Figure 6:**
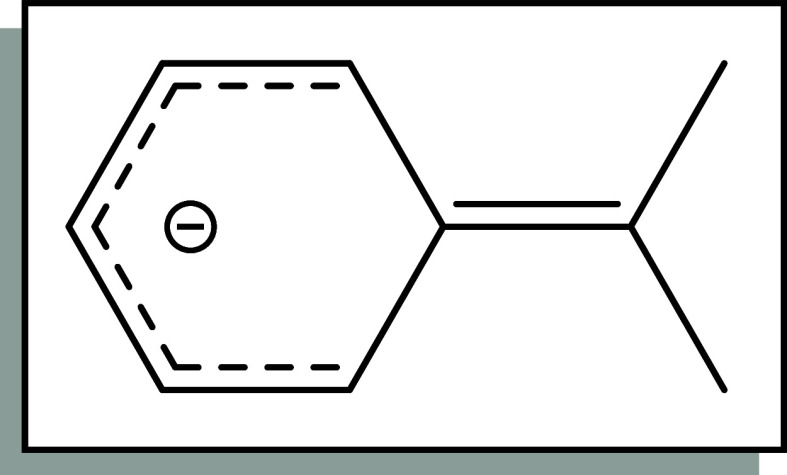
Interpretation of negative charge delocalization in the cumyl anion of complexes **1**-**5** based on bond length analysis.

Applying the method of Alvarez,^60^ we analyzed the hapticity of the interactions between the aromatic rings and alkali-metal cations. As shown in Figure 7, the six different hapticities can easily be mapped onto a six-membered ring, and comparing this figure to the metal positions in our complexes allows a determination of the hapticity of the metal–ligand interaction.

**Figure 7:**
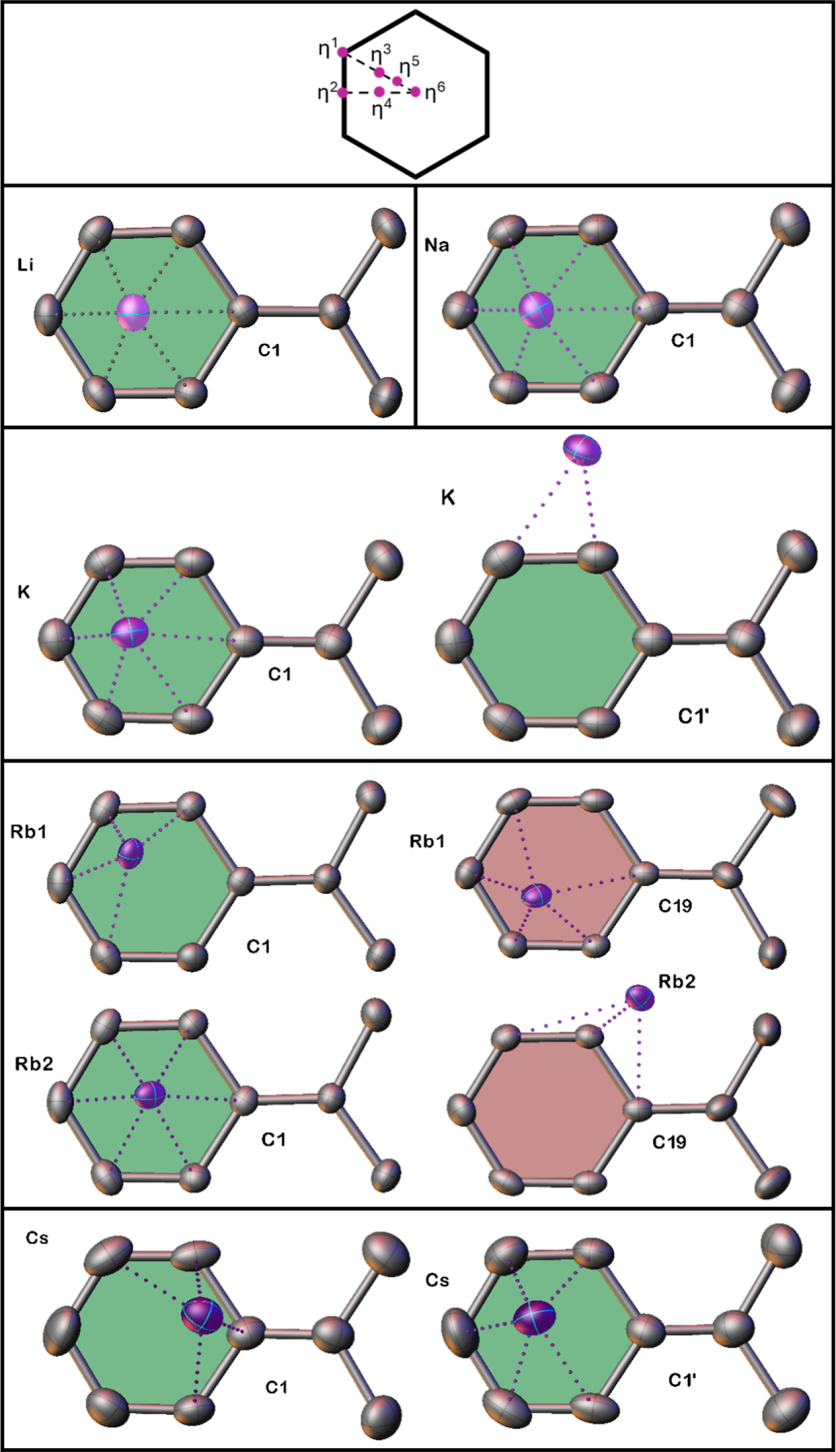
View of metal–ligand relationships in cumyl complexes **1**-**5** taken from directly above the aromatic ring. Red and green rings represent crystallographically independent cumyl ligands.

The discrete monomeric lithium and sodium complexes appear to be approximately η^5^ with the longest interaction in each case being to C_ipso_, in accord with the interpretation of the cumyl anion shown in Figure 6. In contrast, the potassium complex propagates via cumyl-K-cumyl interactions, which according to Figure 7 are best described as η^5^ to the first ring and then η^2^ to one ortho and one meta ring carbon, with the metal lying outside the plane of the six-membered ring in this case. The other supramolecular structure, containing cesium, propagates in a similar way but the greater radius of the cesium cation is reflected via it preferring a η^4^ coordination to the meta-ortho-ipso-ortho’ region of the ring. For the rubidium tetramer, there are two crystallographically independent rubidium cations. The first engages in an η^4^ interaction (to the ring shaded green) and a η^5^ interaction (to the ring shaded red) but both Rb-ring centroid distances are almost identical [3.131(1) and 3.122(1)­Å respectively]. Rb2 appears to favor an η^6^ interaction to the green ring and an η^3^ interaction to the ipso-ortho-meta unit of the red ring, with the metal lying outside the C_6_ unit. This results in vastly different Rb-centroid distances of 2.978(1)­Å and 3.861(1)­Å.

Given the unique nature of the Rb tetramer in this study, we performed DFT calculations to understand the key bonding interactions within this compound. The optimized structure is in good agreement with the experimentally determined structure, including the observed asymmetry in the interactions to cumyl rings. This is further highlighted in the QTAIM analysis, in particular for the η^3^ interaction of the red cumyl ring, with bond paths and critical points centered around the ortho-carbon atom (Figure 8). Bonding interactions within the tetramer are in line with the expected closed-shell ionic interactions with weak van der Waals also highlighted via a NCI plot (See Figure S22).

**Figure 8:**
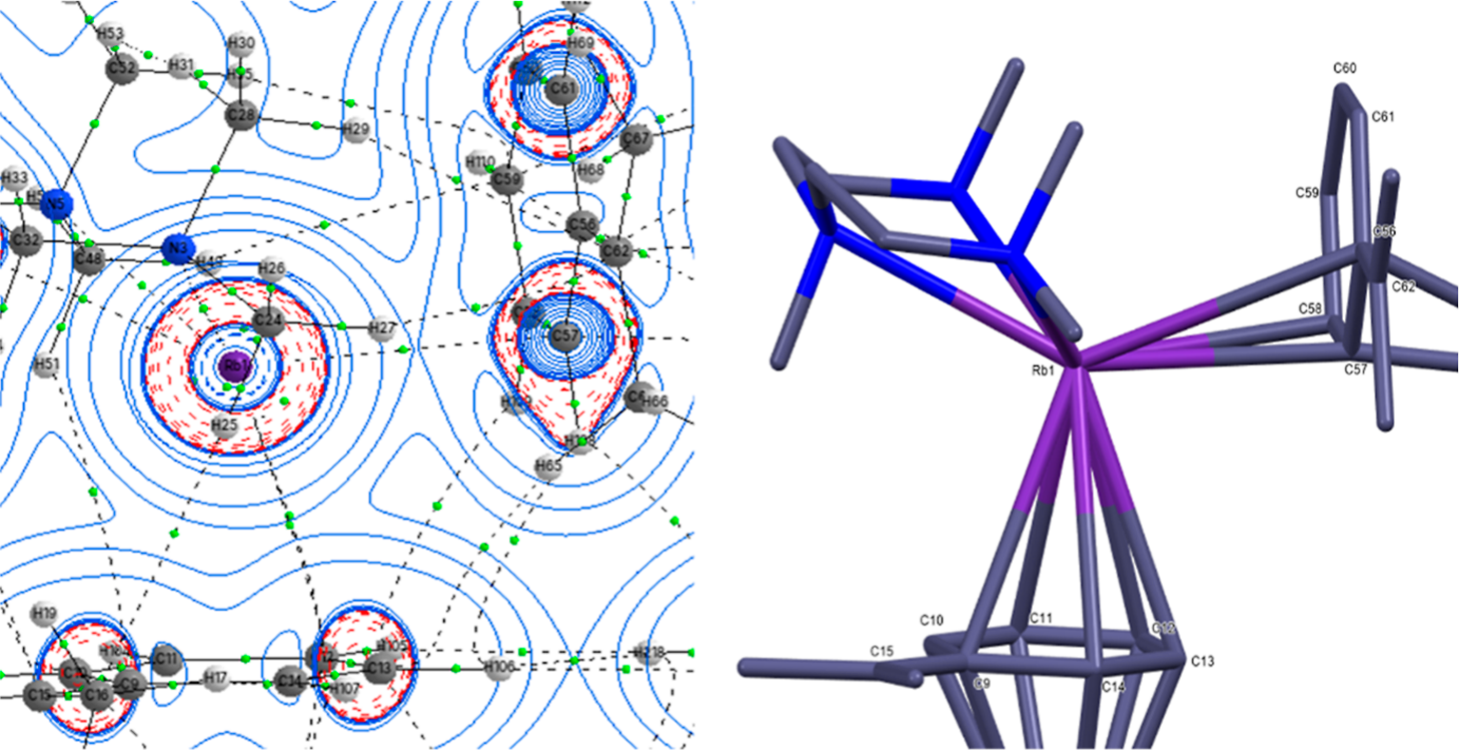
2D contour plot of Laplacian (∇2ρ­(r)) plot across the Rb plane (left) and optimized model structure of **4·PMDETA** (right).

### ^1^H and ^13^C NMR Spectroscopic Study

A ^1^H NMR spectroscopic study of the TMEDA-solvated lithium crystals in C_6_D_6_ solution revealed several resonances in the diagnostic (4.5–8.5 ppm) region of the spectrum. This was filled by six aromatic resonances with three considerably less intense resonances at 6.41/5.65/4.69 ppm (see SI for NMR spectra). The aliphatic region revealed a pair of mutually coupled septets and doublets, indicative of intact iso-propyl groups, and a further singlet. Taken together, and with the help of a COSY NMR experiment, we could deduce that rather than only the two crystallographically characterized isomers (vide supra), there were actually three lithiated complexes present, specifically meta- and para-isopropylphenyl lithium (**1m** and **1p**; that is cumene deprotonated at the meta and para positions of the aromatic ring, respectively) and α,α-dimethylbenzyl lithium (that is where deprotonation of cumene occurs at the lateral α-CH position) in an approximate ratio of 64:32:4 (Scheme 1). Such a product distribution has been seen before by Broaddus who metalated cumene with ^n^BuLi in the presence of TMEDA (followed by carbonation and esterification) and observed a similar meta/para/alpha ratio of approximately 57:30:3 with an additional 10% yield of the ortho-substituted isomer,^24^ one which we find no evidence for in our own analysis. Broaddus also reported virtually no change in these ratios over longer reaction times, suggesting that interconversion between isomers is not taking place in solution. The meta-isomer **1m** displays four aromatic resonances, one singlet for the isolated aromatic CH group between the metal and the ^i^Pr group and three mutually coupled adjacent CH resonances, while the para-isomer **1p** displayed mutually coupled doublets for the meta- and ortho-CH groups. The minor isomer, **1α**, displays three mutually coupled resonances in a 2:2:1 ratio, but these are heavily shielded with respect to a typical benzyl lithium type complex and are more akin to benzyl potassium where the negative charge has been significantly delocalized from the ring-adjacent α-position into the phenyl ring,^11^ suggesting the solid-state structure is maintained in C_6_D_6_ solution. There are only two resonances from the solvating TMEDA at 1.89 and 1.64 ppm, shifted from those of free TMEDA thus confirming coordination to Li, rather than the three sets of two resonances which might be expected as a consequence of having three potential isomers.

The PMDETA solvated sodium complex was also soluble in C_6_D_6_, allowing its study directly by NMR spectroscopy. However, the heavier alkali-metal complexes (K–Cs) were insoluble, so one equivalent of PMDETA was added to a C_6_D_6_ suspension and this was filtered, in an attempt to replicate the 1:1 stoichiometric ratio seen in the solid-state structures. However, this did not sufficiently dissolve all the organometallic compound and so a considerable excess of PMDETA swamped their spectra, meaning the coordination of it to the metal could not be definitively determined, although the resonances of the organoanion were clear.

In contrast to the solution complexity of the NMR spectra of the lithium compounds, the heavier alkali-metal complexes (**2**, Na; **3**, K; **4**, Rb; **5**, Cs) each show only one set of resonances, specifically the 2:2:1 mutually coupled set between 4.6 and 6.7 ppm and a singlet about 2 ppm (see Table 2 for details), suggesting that these heavier alkali-metal bases exclusively metalate at the alpha CH position. The similarities between the spectra supports a common metal–ligand bonding motif, in accord with the solid-state structures and in stark contrast to the alkali-metal benzyl family of complexes where the metal sequentially shifts from a σ-bond to the deprotonated carbon atom (M = Li) with increasing π-bond character to the aromatic ring (M = K). The chemical shifts of the ring CH units would favor the latter scenario. In the cases of the lighter Na and K congeners the PMDETA donor resonances suggest coordination to the metal, with the heavier congeners (Rb, Cs) showing resonances coinciding with those of free PMDETA. This presumed loss of PMDETA from the Rb and Cs coordination spheres is not surprising since the solvent is an arene, the π-system of which is well-known to engage with these softer metals.^61–65^ The ^13^C NMR data (Table S2) were also studied with assignments of the relevant resonances being straightforward, with the exception of **1** due to the isomeric nature of the crude product. The resonances of **1** could be enhanced by manually separating the colorless (ring-metalated) and red (laterally metalated) crystals allowing for easier assignment.

**Table 2:** Selected ^1^H NMR Data (ppm, 400.13 MHz) of Alkali-Metal Cumyl Complexes in C_6_D_6_

	ortho	meta	para	CH	CH_3_
**1m**	8.23/8.14	7.38	7.13	3.04	1.47
**1p**	8.29	7.29	-	2.94	1.39
**1α**	5.65	6.41	4.69	-	1.93
**2**	5.77	6.63	4.85	-	2.04
**3**	5.59	6.50	4.83	-	1.99
**4**	5.62	6.57	4.97	-	1.97
**5**	5.38	6.40	4.94	-	1.91
cumene	7.10	7.18	7.07	2.71	1.13

### DOSY NMR Study

Given the structural variety within this set of cumyl alkali-metal complexes, we next turned to ^1^H DOSY (diffusion-ordered) NMR spectroscopy to gain insight into their solution state aggregation. **1**p was studied through the isolated powder, while **1m** and **1α** used recrystallized material which had some of the dominant **1m** isomer removed by hand to enhance the ratio and concentration of **1α**. While the resonances of the different anionic isomers in these mixtures were well-defined, the overlapping nature of the TMEDA resonances rendered them uninformative in this particular analysis. Some caution is required in interpreting the results, as is common in DOSY studies, with the most uncertainty with the para-isomer data due to the lack of a crystal structure to support the data from this solution investigation. The four remaining heavier alkali-metal complexes could all be analyzed straightforwardly, in C_6_D_6_ solution, using the dissipated spheres and ellipsoids (DSE) external calibration curves developed by Stalke.^66,67^

The predicted molecular weights calculated for the lithium complexes (**1m**, **1p** and **1α**) were 431, 525, 219 respectively, when based upon the cumyl resonances (see Table 3 for details of diffusion coefficients and resulting predicted molecular weights). The calculated molecular weights for both the meta- and alpha-isomers are close to those of their aggregation states observed in the solid-state, with **1m** forming a dimer ([^i^PrC_6_H_4_Li·TMEDA]_2_, 484 g mol^–1^, +12% error) and **1α** forming a monomer, CumylLi·TMEDA (242 g mol^–1^, +11%). The data suggests that **1p** also forms a dimer in solution (525 g mol^–1^, −8%), however the molecular weight is overestimated here rather than underestimated, emphasizing the speculative nature of these interpretations and demonstrating the difficulty of utilizing this technique upon isomeric mixtures.

**Table 3:** Diffusion Coefficients From 2D ^1^H DOSY NMR Spectra of Complexes **1**-**5** in C_6_D_6_

	**1m** ^a^	**1p** ^b^	**1α** ^a^	**2**	**3**	**4**	**5**
mean D_Cumyl_ (*x* 10 ^–10^) (m^2^ s^–1^)	6.4600	6.4975	11.1450	8.4650	4.0575	4.2675	3.7300
mean *D*_Donor_ (*x* 10 ^–10^) (m^2^ s^–1^)	7.6600	7.3300	7.6600	8.3200	7.6033	11.1750	11.3750
*D*_standard_ (*x* 10^–9^)	1.60	1.82	1.60	1.81	1.60	1.66	1.63
standard	TMS	TMS	TMS	TMS	TMS	C_6_D_6_	C_6_D_6_
donor	TMEDA	TMEDA	TMEDA	PMDETA	PMDETA	PMDETA	PMDETA
MW_cumyl_ (g mol^–1^)	431	525	219	340	909	811	978
MW_donor_ (g mol^–1^)	-	-	-	350	331	173	169

a Handpicked crystalline samples of the desired isomer were used for this study data.

b The powder mixture sample was used for this study data.

For the sodium (**2**), potassium (**3**), rubidium (**4**) and cesium (**5**) complexes the estimated MW’s based on cumyl resonances are, 340, 909, 811, and 978 g mol^–1^ respectively (see Table 3). Using the PMDETA resonances, the molecular weights could be estimated as 350, 331, 173, and 169 g mol^–1^ for **2**-**5** respectively. Focusing on the PMDETA resonances first, the rubidium (0% error) and cesium (+6% error) complexes give values remarkably close to that of the metal-free triamine (173 g mol^–1^), suggesting its likely decoordination in benzene solution. This aligns well with the affinity of Rb and Cs for soft, π-electron density such as that provided by an aromatic solvent over the hard nitrogen atoms of PMDETA. For potassium, the value is intermediate between that of free PMDETA and the values of the cumyl anion, indicative of a possible coordination-decoordination event in solution. Finally, the value for the PMDETA molecule in the sodium complex (350) is reasonably close to that of the cumyl anion (340), suggesting a tightly bound PMDETA molecule. Taken together, these results are thus consistent with a solution formula of monomeric CumylNa·PMDETA (replicating the solid-state structure, MW 315 g mol^–1^, −7%), [CumylRb]_4_ (819 g mol^–1^, +1%) and [CumylCs]_4_ (1008 g mol^–1^, +3%) for **2**, **4** and **5** respectively. Results for potassium complex **3** are rather less clear-cut, with a PMDETA rich dinuclear complex [CumylK]_2_[PMDETA]_3_ (836 g mol^–1^, −8%), trimeric [CumylK·PMDETA]_3_ (995g mol^–1^, +9%), and unsolvated hexamer [CumylK]_6_ (950 g mol^–1^, +5%) all lying within reasonable experimental error. To the best of our knowledge there are no precedents for similar aggregates in the literature and so these suggestions must be treated with caution, although Hevia and Gessner also reported solution aggregation of PMDETA-solvated 2-naphthylmethyl potassium as larger than dimeric by ^1^H DOSY spectroscopy.^5^

## Experimental Section

Caution! Extreme care should be taken both in the handling of the cryogen liquid nitrogen and its use in the Schlenk line trap to avoid the condensation of oxygen from air.

Caution! *n*-butyllithium is pyrophoric. It must be handled using proper needle and syringe techniques.^68^ All manipulations were performed on the smallest practical scale following the procedures described in the experimental section.

Caution! All cumyl species synthesized should be treated as pyrophoric. They must be handled using proper Schlenk and air sensitive techniques.^68^ All manipulations were performed on the smallest practical scale following the procedures described in the experimental section.

### General Experimental Information

Due to the air- and moisture-sensitivity of these organoalkali metal compounds, all synthetic procedures were performed under a dry N_2_ atmosphere using standard Schlenk techniques or in a glovebox under a recirculating Ar atmosphere. Prior to use, glassware was predried in an oven at 150 °C, then heated with a heat gun under vacuum. C_6_D_6_ (purchased from Apollo Scientific or Sigma-Aldrich) was stored in the glovebox over activated molecular sieves (4 Å). Hexane (purchased from Sigma-Aldrich) was dried in a solvent purification system (Innovative Technology, PS-Micro), degassed, and stored under an inert atmosphere over activated 4 Å molecular sieves. Cumene (purchased from Acros Organics) was dried over sodium and benzophenone, distilled under a N_2_ atmosphere, and stored over activated 4 Å molecular sieves prior to use. *N*,*N*,*N′*,*N′*-tetramethylethylenediamine (TMEDA, purchased from Alfa Aesar) and *N*,*N*,*N′*,*N″*,*N″*-pentamethyldiethylenetriamine (PMDETA, purchased from Sigma-Aldrich) were both dried over calcium hydride powder, distilled under a N_2_ atmosphere, and stored over activated 4 Å molecular sieves prior to use.

^n^BuLi and KO^t^Bu were purchased from Sigma-Aldrich and used as received. ^n^BuNa^69^ and MO^t^Am (M = Rb, Cs)^70^ were synthesized according to known literature procedures.

^1^H, ^13^C­{^1^H}, COSY, and DOSY NMR spectra were recorded on an AV300 or AV400 MHz spectrometer. Chemical shifts (δ in ppm) in the ^1^H and ^13^C NMR spectra were referenced to the residual signals of the deuterated solvents. Common abbreviations have been used to describe signal multiplicities: s (singlet), d (doublet), t (triplet), q (quartet), dd (doublet of a doublet), m (multiplet) and br (broad). DOSY data were obtained through analysis of the PMDETA ligated crystals of **2·PMDETA**, **3·PMDETA**, **4·PMDETA**, and **5·PMDETA**, and a hand-picked mixture of the TMEDA ligated crystals for **1m·TMEDA** and **1α·TMEDA** to ensure adequate concentrations for analysis. Flame-dried J Young’s NMR tubes were used for all DOSY studies.

Several attempts were made to confirm bulk purity via elemental analysis but consistent results could not be obtained. NMR spectra are provided (see Supporting Information) to confirm proof of bulk purity.

The complete crystallographic data of the new crystalline compounds can be found in Table S3.

### Synthesis of 1·TMEDA

To a stirring solution of TMEDA (0.16 mL, 1 mmol) in cumene (5 mL) at 0 °C was added ^n^BuLi (0.7 mL, 1 mmol, 1.51 M in hexanes) dropwise via syringe. The solution was allowed to warm to room temperature and slowly produced a deep red solution over 10 min. The solution was left to stir overnight. The volatiles were removed in vacuo, giving a deep red sticky solid (crude yield, 96 mg, 0.40 mmol, 37%). NMR analysis suggested three isomeric complexes were present in solution, assigned as the alpha-, meta- and para-lithiated complexes, in a 4:64:32 ratio.

#### 1α·TMEDA

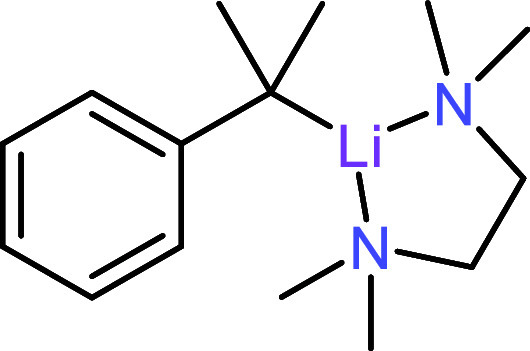



^1^H NMR (400 MHz, C_6_D_6_): δ 6.41 (dd, *J* = 9.2, 6.4 Hz, 2H, C_meta_–H), 5.65 (dd, *J* = 9.0, 1.4 Hz, 2H, C_ortho_–H), 4.69 (t, *J* = 6.4 Hz, 1H, C_para_–H), 1.93 (s, 6H, C_Me_–H), 1.89 (s, TMEDA, NMe_2_), 1.64 (s, TMEDA, NCH_2_).

^13^C NMR (101 MHz, C_6_D_6_) δ 134.9 (C_ipso_), 128.6 (C_ortho_), 105.1 (C_meta_), 85.6 (C_para_), 70.6 (C_α_), 56.6 (CH_2_ of TMEDA), 46.0 (CH_2_ of TMEDA), 20.8 (C_Me_)

#### 1m·TMEDA

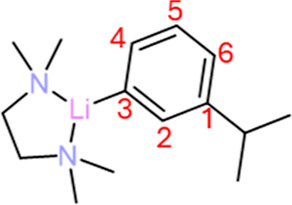



^1^H NMR (400 MHz, C_6_D_6_): δ 8.23 (s, 1H, C_2_–H), 8.14 (d, *J* = 6.5 Hz, 1H, C_6_–H), 7.38 (t, *J* = 7.0 Hz, 1H, C_5_–H), 7.13 (d, *J* = 7.5 Hz, 1H, C_4_–H), 3.04 (sept, *J* = 6.9 Hz, 1H, C_α_–H), 1.89 (s, TMEDA, NMe_2_), 1.64 (s, TMEDA, NCH_2_), 1.47 (d, *J* = 6.9 Hz, 6H, C_Me_–H).

^13^C NMR (101 MHz, C_6_D_6_): δ 187.4 (C_3_), 143.5 (C_1_), 143.1 (C_2_), 142.6 (C_6_), 125.1 (C_5_), 121.8 (C_4_), 56.6 (CH_2_ of TMEDA), 46.0 (CH_2_ of TMEDA), 35.5 (C_α_), 25.0 (C_Me_)

#### 1p·TMEDA

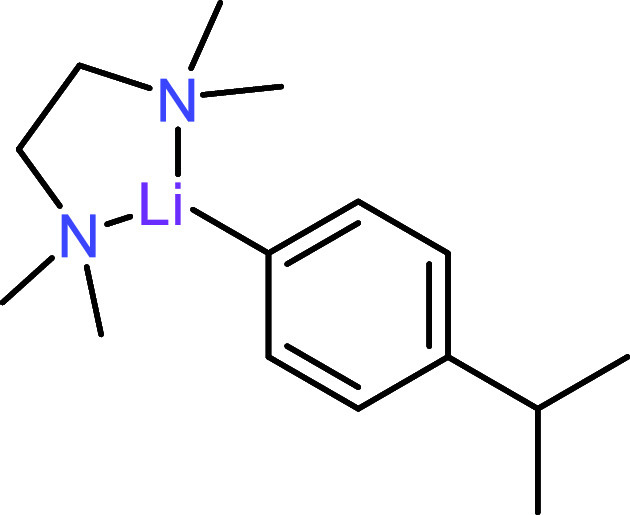



^1^H NMR (400 MHz, C_6_D_6_): δ 8.29 (d, *J* = 7.5 Hz, 2H, C_ortho_–H), 7.29 (d, *J* = 7.8 Hz, 2H, C_meta_–H), 2.94 (sept, *J* = 6.9 Hz, 1H, C_α_–H), 1.89 (s, TMEDA, NMe_2_), 1.64 (s, TMEDA, NCH_2_), 1.39 (d, *J* = 6.9 Hz, 6H, C_Me_–H).

^13^C NMR (101 MHz, C_6_D_6_): δ 182.9 (C_para_), 145.0 (C_ortho_), 143.7 (C_ipso_) 123.5 (C_meta_), 56.6 (TMEDA CH_3_), 46.0 (TMEDA CH_2_), 34.8 (C_α_), 25.3 (C_Me_)

Addition of ^n^hexane to the crude **1·TMEDA** product mixture afforded a transparent, deep red solution, which was stored in the glovebox at – 20 °C. After 10 days, two distinct types of crystals had formed, namely colorless blocks of **1α·TMEDA** and deep red rods of **1m·TMEDA**.

### Synthesis of Sodium Complex 2·PMDETA

PMDETA (0.420 mL, 2.01 mmol) was added to a stirring suspension of ^n^BuNa (160 mg, 2.0 mmol) in ^n^hexane (5 mL), forming a yellow solution. The solution was cooled to 0 °C, and cumene (0.3 mL, 2 mmol) was added dropwise. Over the course of the addition the solution changed color from yellow to deep red. Warming the solution to room temperature and stirring for 2 h gave a deep red precipitate. The mixture was filtered and the collected solid was washed with aliquots of hexane (3 × 5 mL) and dried in vacuo to afford a dark red powder (326 mg, 1.03 mmol, 52%). The product was stored in the glovebox freezer at – 30 °C.

^1^H NMR (400 MHz, C_6_D_6_): δ 6.63 (dd, *J* = 9.0, 6.4 Hz, 2H, C_meta_–H), 5.77 (dd, *J* = 9.0, 1.4 Hz, 2H, C_ortho_–H), 4.85 (t, *J* = 6.4 Hz, 1H, C_para_–H), 2.04 (s, 6H, C_Me_–H), 1.90–1.71 (m, 29H, PMDETA).

^13^C NMR (101 MHz, C_6_D_6_): δ 136.9 (C_ipso_), 129.4 (C_ortho_), 104.8 (C_meta_), 86.7 (C_para_), 67.6 (C_α_), 57.4 (PMDETA CH_2_), 55.1 (PMDETA CH_2_), 45.4 (PMDETA N­(CH_3_)_2_), 43.3 (PMDETA NCH_3_), 21.4 (C_Me_).

### Synthesis of Potassium Complex 3

Cumene (0.3 mL, 2 mmol) was added to a stirring suspension of KO^t^Bu (220 mg, 2.0 mmol) in ^n^hexane (10 mL). The suspension was cooled to 0 °C, to which ^n^BuLi (1.4 mL, 2.2 mmol, 1.6 M in hexanes) was added dropwise. After the addition, the suspension changed color from colorless to a deep red. Next, the suspension was warmed to room temperature and stirred for 1 h. The mixture was filtered and the collected solid was washed with aliquots of hexane (3 × 5 mL) and dried in vacuo to afford a dark red powder (292 mg, 1.83 mmol, 94%). The product was stored in the glovebox.

For the SCXRD study, one molar equivalent of PMDETA was added to a suspension of **3** in cumene, affording a transparent, deep red solution, which was stored in the glovebox at – 20 °C. After one-week, deep red blocks of **3·PMDETA** suitable for SCXRD analysis formed.

For the NMR study, C_6_D_6_ (0.5 mL) was added to a vial charged with **3** (11 mg, 73 μmol), with no visible dissolution. One molar equivalent of PMDETA (15.3 μL, 73.3 μmol) was then added to the vial affording a suspension with a deep red supernatant. The suspension was filtered and loaded into a J Young’s NMR tube, thus leading to the excess of PMDETA observed in the spectrum.

^1^H NMR (400 MHz, C_6_D_6_): δ 6.50 (dd, *J* = 8.9, 6.4 Hz, 2H, C_meta_–H), 5.59 (dd, *J* = 9.0, 1.3 Hz, 2H, C_ortho_–H), 4.83 (t, *J* = 6.3 Hz, 1H, C_para_–H), 2.22–2.05 (m, 30H, PMDETA), 1.99 (s, 6H, C_Me_–H).

^13^C NMR (101 MHz, C_6_D_6_): δ 136.9 (C_ipso_), 130.4 (C_ortho_), 105.9 (C_meta_), 88.6 (C_para_), 68.5 (C_α_), 57.7 (PMDETA CH_2_), 56.6 (PMDETA CH_2_), 45.5 (PMDETA N­(CH_3_)_2_), 41.8 (PMDETA NCH_3_), 21.1­(C_Me_).

### Synthesis of Rubidium Complex 4

RbO^t^Am (174 mg, 1.01 mmol) was added to cumene (5 mL), forming a solution. The solution was cooled to 0 °C, and ^n^BuLi (0.63 mL, 1.0 mmol, 1.6 M in hexanes) was added dropwise via syringe. Over the course of the addition the solution turned from a yellow color to a deep red suspension, with a free moving solid. The suspension was warmed to room temperature and stirred for 1 h, following which the solid merged into a large thick mass at the bottom of the Schlenk tube. The mixture was filtered and the solid was washed vigorously with hexane (3 × 5 mL) and dried in vacuo to afford a dark red powder (89 mg, 0.43 mmol, 43%). The product was stored in the glovebox.

For the SCXRD study, one molar equivalent of PMDETA was added to a suspension of **4** in cumene, affording a transparent, deep red solution, which was stored in the glovebox at – 20 °C. After one-week, deep red block crystals of **4·PMDETA** suitable for SCXRD analysis formed.

For the NMR study, C_6_D_6_ (0.5 mL) was added to a vial charged with **4** (15 mg, 73 μmol), with no visible dissolution. One molar equivalent of PMDETA (15.3 μL, 73.3 μmol) was then added to the vial affording a suspension with a deep red supernatant. The suspension was filtered and loaded into a J Young’s NMR tube, thus leading to the excess of PMDETA observed in the spectrum.

^1^H NMR (400 MHz, C_6_D_6_): δ 6.57 (dd, *J* = 8.9, 6.4 Hz, 2H, C_meta_–H), 5.62 (dd, *J* = 8.9, 1.4 Hz, 2H, C_ortho_–H), 4.97 (t, *J* = 6.4 Hz, 1H, C_para_–H), 2.48–2.09 (m, PMDETA), 1.97 (s, 6H, C_Me_–H).

^13^C NMR (101 MHz, C_6_D_6_): δ 137.7 (C_ipso_), 130.7 (C_ortho_), 106.4 (C_meta_), 89.7 (C_para_), 66.9 (C_α_), 58.3 (PMDETA CH_2_), 57.0 (PMDETA CH_2_), 46.0 (PMDETA N­(CH_3_)_2_), 43.0 (PMDETA NCH_3_), 21.3 (C_Me_).

### Synthesis of Cesium Complex 5

Cumene (0.28 mL, 2.0 mmol) was added to a stirring suspension of CsO^t^Am (442 mg, 2.01 mmol) in ^n^hexane (10 mL). The suspension was cooled to 0 °C, and ^n^BuLi (1.25 mL, 2.01 mmol, 1.6 M in hexanes) was added dropwise via syringe. Immediately upon the addition a deep red free moving precipitate formed. The suspension was stirred for 1 h. The mixture was filtered and the solid was washed with hexane (3 × 5 mL) and dried in vacuo to afford a dark red powder (430 mg, 1.27 mmol, 56%). The product was stored in the glovebox.

For the SCXRD study, one molar equivalent of PMDETA was added to a suspension of **5** in cumene, affording a transparent, deep red solution, which was stored in the glovebox at – 20 °C. After one-week, deep red block crystals of **5·PMDETA** suitable for SCXRD analysis formed.

For the NMR study, C_6_D_6_ (0.5 mL) was added to a vial charged with **5** (20 mg, 79 μmol), with no visible dissolution. One molar equivalent of PMDETA (16.6 μL, 79.3 μmol) was added to the vial affording a suspension with a deep red supernatant. The suspension was filtered and loaded into a J Young’s NMR tube, thus leading to the excess of PMDETA observed in the spectrum.

^1^H NMR (400 MHz, C_6_D_6_): δ 6.40 (dd, *J* = 8.8, 6.5 Hz, 2H, C_meta_–H), 5.38 (dd, *J* = 9.0, 1.3 Hz, 2H, C_ortho_–H), 4.94 (t, *J* = 6.5 Hz, 1H, C_para_–H), 2.42–2.08 (m, PMDETA), 1.91 (s, 6H, C_Me_–H).

^13^C NMR (101 MHz, C_6_D_6_): δ 138.8 (C_ipso_), 131.6 (C_ortho_), 107.0 (C_meta_), 91.2 (C_para_), 68.0 (C_α_), 58.1­(PMDETA CH_2_), 56.8 (PMDETA CH_2_), 45.8 (PMDETA NCH_3_), 42.6 (PMDETA N­(CH_3_)_2_), 21.3 (C_Me_).

## Conclusions

In this work we have synthesized as well as crystallographically and spectroscopically characterized six new alkali metal cumyl complexes, five of which contain a different metal from lithium to cesium, supported by the Lewis base TMEDA (for Li) or PMDETA (Na–Cs), as well an isomeric second structure for lithium also with TMEDA. Synthetically, the reactions involving the heavier alkali metals proved more selective than those of their lighter lithium counterparts as they produced one isolable product, namely the α,α-dimethylbenzylmetal compounds, whereas with lithium, a mixture was formed containing products metalated at phenyl ring sites as well as the α,α-dimethylbenzyllithium complex. The solid-state structural results can be subdivided into four distinct categories. Most understandably the meta-deprotonated lithium complex is distinctly different from its alpha-deprotonated isomer having a σ-bonded dimeric, buckled (Li–C)_2_ ring with terminal TMEDA ligation. In contrast, in this alpha-deprotonated isomer the lithium engages with the π face of the phenyl ring in an η^5^-manner, like its sodium counterpart with the only different significant feature being the capping of the metal cation, which is two-coordinate by TMEDA for Li and three-coordinate by PMDETA for Na. Both the potassium and cesium complexes adopt similar one-dimensional infinite zigzag chain structures with the only important distinction between them being the extent of hapticity of their π-phenyl ring bonding to the respective metal cations. The standout unique structure is that of the rubidium complex which cyclizes into a π-phenyl bonded discrete tetramer, with external PMDETA ligands completing the coordination of the Rb^+^ cations.

Such structural comparisons covering the whole alkali metal set are of increasing significance as they can be useful guides to aid mechanistic understanding in catalytic applications for example providing model structures for computational studies. Heavier, softer s-block metals propensity to engage in noncovalent interactions can give rise to novel outer sphere reaction mechanisms for example in catalytic alkene insertion reactions^71^ in contrast to more traditional direct σ-bond insertion routes.^72^

## Supplementary Material



## Notes

Supplementary Introductory Information, NMR spectra, computational details, X-ray crystallographic data and DOSY data (PDF)
