# Supplementary material for: Comparisons and Contrasts in a Complete Set of Alkali Metal Cumyl Structures
Source: Inorg Chem. 2026 Mar 27;65(14):7993–8004. doi: 10.1021/acs.inorgchem.6c00468 (PMC13080972; doi:10.1021/acs.inorgchem.6c00468)
Supplement: Supplementary file 1 [file ic6c00468_si_001.pdf]

Electronic Supporting Information for

Comparisons and Contrasts in a Complete Set of Alkali Metal Cumyl Structures

Paul D.L. Ferguson, David Anderson, Eva Hevia, Thomas M. Horsley Downie, Alan R. Kennedy, Stuart D. Robertson,* Catherine E. Weetman, Robert E. Mulvey*

Department of Pure and Applied Chemistry, University of Strathclyde, 295 Cathedral Street, Glasgow, G1 1XL, U.K.

Department für Chemie und Biochemie, Universität Bern, Freiestrasse 3, 3012 Bern, Switzerland

r.e.mulvey@strath.ac.uk; stuart.d.robertson@strath.ac.uk

Contents

**1**	**Supplemental Introductory Information**	**S2**
**2**	**Characterisation of Compounds**	**S3**
	2.1 Cumyllithium·TMEDA (**1·TMEDA**)	S3
	2.2 *Alpha*-cumylsodium (**2·PMDETA**)	S8
	2.3 *Alpha*-cumylpotassium (**3**)	S9
	2.4 *Alpha*-cumylrubidium (**4**)	S10
	2.5 *Alpha*-cumylcesium (**5**)	S11
**3**	**Computational Studies**	**S13**
**4**	**X-ray Crystallographic Data (SCXRD)**	**S20**
**5**	**DOSY Spectroscopy**	**S22**
	5.1 DOSY of **1·TMEDA**	S22
	5.2 DOSY of **2·PMDETA** crystals	S24
	5.3 DOSY of **3·PMDETA** crystals	S25
	5.4 DOSY of **4·PMDETA** crystals	S26
	5.5 DOSY of **5·PMDETA** crystals	S27
**6**	**References**	**S28**

1. Supplemental Introductory Information

Table S1 Summary of published metalation reactions of cumene with various alkali-metal bases

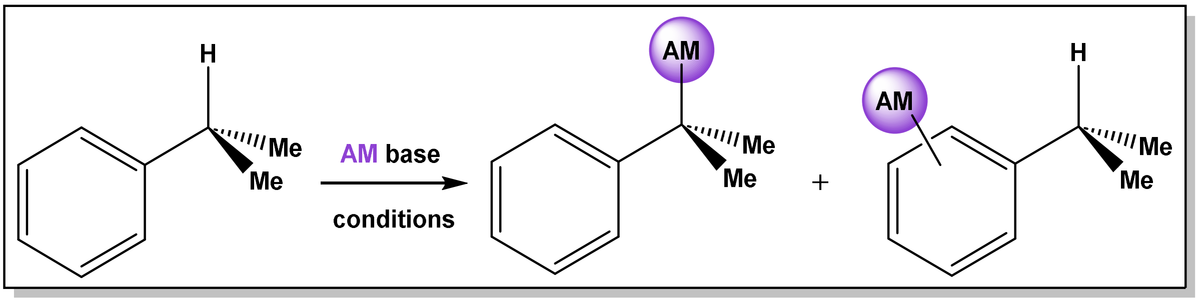

Entry	Base	Solvent	T (°C)	Time	Add.	Yield (%)	ortho	meta	para	alpha	ref
1	EtNa	cumene	rt	12 h	-	41	-	-	-	100	1
2	AmNa	cumene	rt	o/n	-	[a]	'some'[d]	-	'largely'[d]	-	2
3	EtK	cumene	rt	10 h	-	[a]	-	-	-	'some'[d]	3
4	AmNa/ NaO^{i}Pr	cumene	rt	4 h	-		11.5	-	88.5	-	4
5	EtK	cumene	rt	8 d	-	31[b]	9	48.5	29.5	13	5
6	EtK	cumene	85	1 h	-	18.5[b]	11.5	44.5	25	19	5
7	^{n}PrK	cumene	90	4 h	-	25.6[b]	14	43	24	19	5
8	^{n}AmK	cumene	rt	8 d	-	36.5[b]	9.5	45.5	32	13	5
9	^{n}AmNa	cumene	rt	10 d	-	34.3[b]	3	49.5	46.5	1	5
10	^{n}AmNa	cumene	rt	20 h	-	[a]	-	55.6	41.8	2.6	6
11	^{n}AmNa	cumene	rt	20 h	-	[a]	-	56	42.8	1.2	6
12	^{n}AmK	cumene	rt	20 h	-	[a]	-	4.5	7.5	88	6
13	^{n}AmK	cumene	rt	20 h	-	[a]	-	-	8.4	91.6	6
14	^{n}AmK	heptane	rt	20 h	-	[a]	-	-	-	100	6
15	^{n}AmK	heptane	rt	3 h	-	[a]	-	39	19	42	6
16	^{n}BuLi	cumene	30	2 h	TMEDA	[a]	10	57	30	3	7
17	^{n}BuLi	cumene	30	24 h	TMEDA	[a]	8	59	30	3	7
18	^{n}AmNa	octane	rt	1 h	TMEDA	[c]	3	57	33	7	8
19	^{n}AmNa	octane	rt	24 h	TMEDA	65[b]	-	3	2	95	8
20	^{n}AmNa	octane	rt	24 h	-	40[b]	-	55	45	-	8
21	^{n}BuLi	MCH	rt	1 h	TMEDA	[a]	'small amount'[d]	'major'[d]		'trace'[d]	9
22	^{n}BuLi	MCH	elevated	1 h	TMEDA	[a]					9
23	^{n}BuLi	CH	rt	1 h	TMEDA	[a]					9
24	^{n}BuLi	CH	elevated	1 h	TMEDA	[a]					9
25	^{n}BuLi	THF	rt	1 h	TMEDA	No reaction					9
26	PhNa	cumene	112	1.5 h	-	35[b]	-	-	-	100	10
27	K	cumene	75	3 h	18-c-6	78[b]	-	-	-	100	10
28	NpNa		-40	4 h	PMDETA	80	-	67	33	-	11
29	NpNa		rt	o/n	PMDETA	58	-	-	-	100	11

[a]: not reported; [b] ratio calculated for organic products upon workup; [c] no yield as an aliquot was taken from the reaction which was left for longer; [d] no specific details were given so original manuscript description is provided; MCH = methylcyclohexane, CH = cyclohexane, 18-c-6 = 18-crown-6.

2. Characterisation of Compounds

Table S2 Selected $^{13}C\{^1H\}$ NMR data (ppm, 101 MHz) of alkali-metal cumyl complexes in C_6D_6.

	ortho	*meta*	*para*	*ipso*	Cα	CH_3
**1m**	143.1/142.6	187.4/125.1	121.8	143.5	35.5	25.0
**1p**	145.0	123.5	182.9	143.7	34.8	25.3
**1α**	128.6	105.1	85.6	134.9	70.6	20.8
**2**	129.4	104.8	86.7	136.9	67.6	21.4
**3**	130.4	105.9	88.6	136.9	68.5	21.1
**4**	130.7	106.4	89.7	137.7	66.9	21.3
**5**	131.6	107.0	91.2	139.8	68.0	21.3
cumene	128.7	126.7	126.2	148.9	34.5	24.2

2.1. Cumyllithium·TMEDA (**1·TMEDA**)

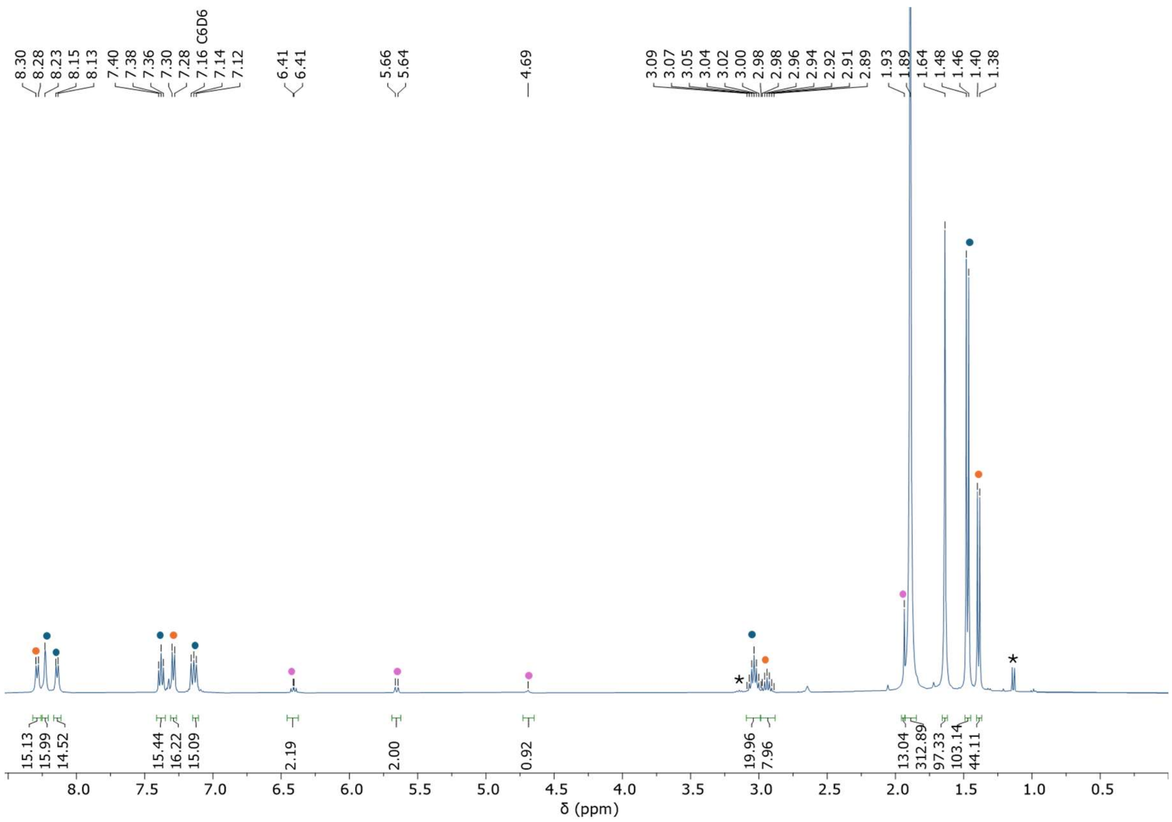

Figure S1 ^1H NMR spectrum (400 MHz, C_6D_6) of **1·TMEDA**, orange circles = **1p·TMEDA**, blue circles = **1m·TMEDA**, and purple circles = **1α ·TMEDA** (* = free cumene)

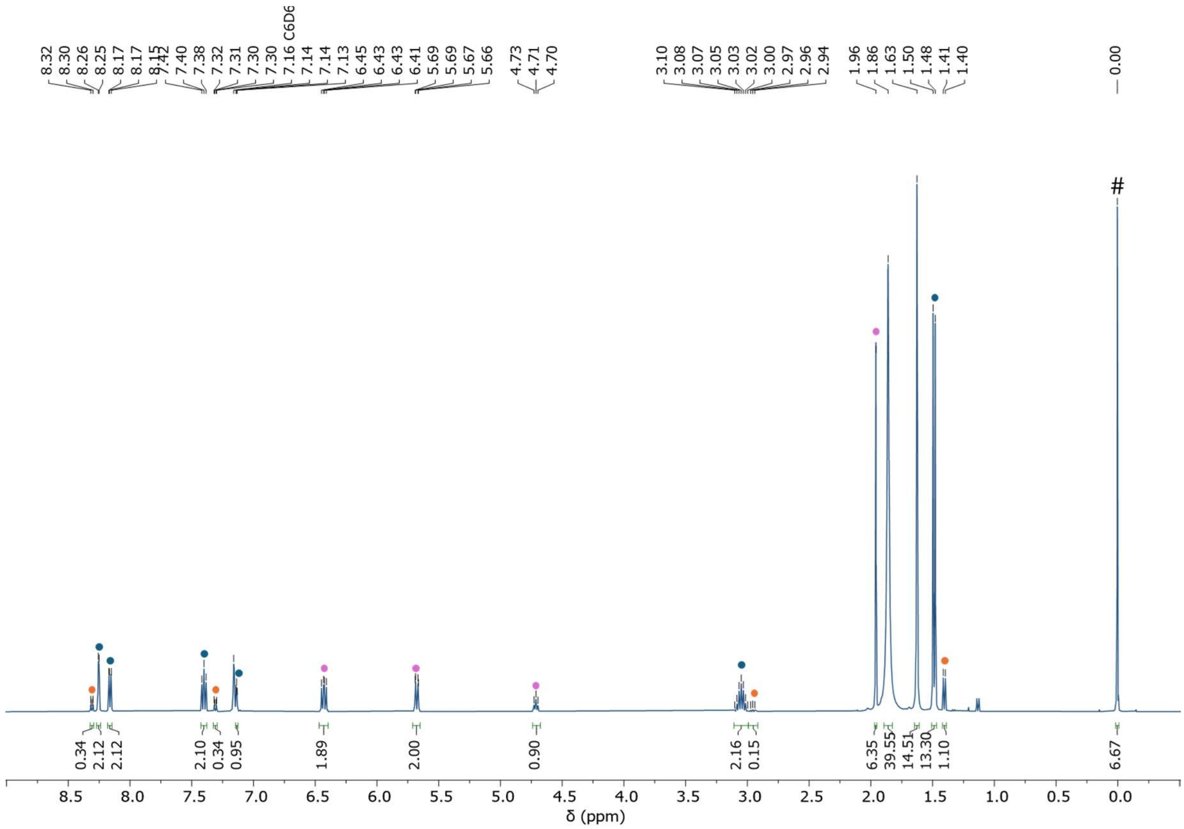

Figure S2 ^{1}H NMR spectrum (400 MHz, C_6D_6) of **1m·TMEDA** and **1α ·TMEDA** crystals. Orange circles = **1p·TMEDA**, blue circles = **1m·TMEDA**, and purple circles = **1α ·TMEDA** (# = TMS)

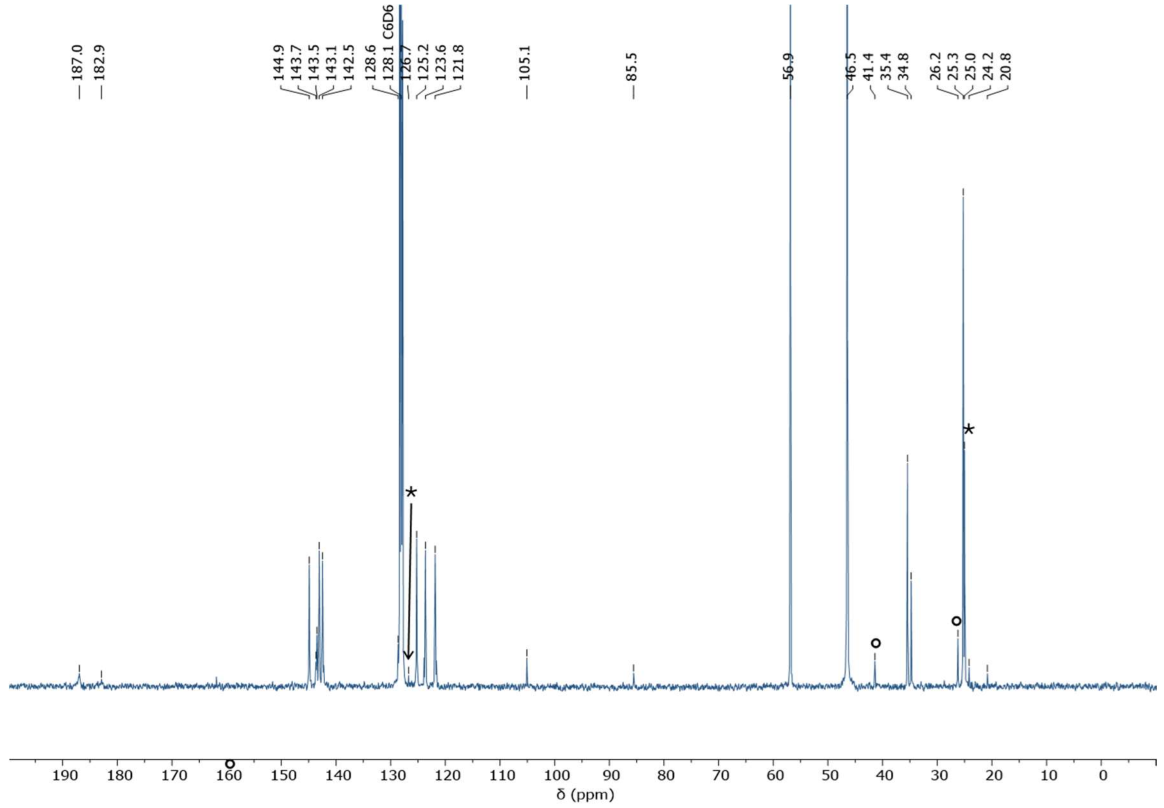

Figure S3 ^{13}C{^{1}H} NMR spectrum (101 MHz, C_6D_6) of isolated crude **1·TMEDA** product (* = free cumene, ° = impurities)

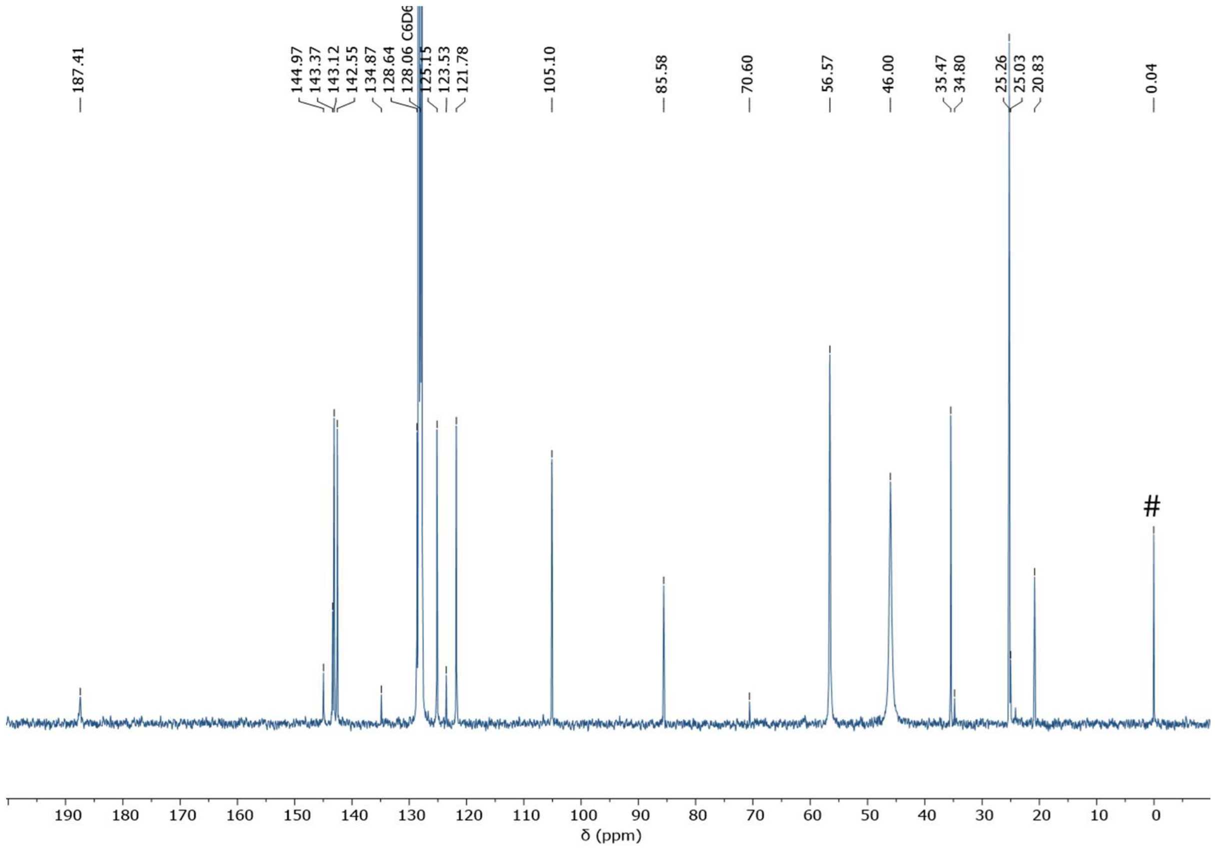

Figure S4 $^{13}C\{^{1}H\}$ NMR spectrum (101 MHz, C_6D_6) of **1m·TMEDA** and **1α ·TMEDA** crystals (# = TMS)

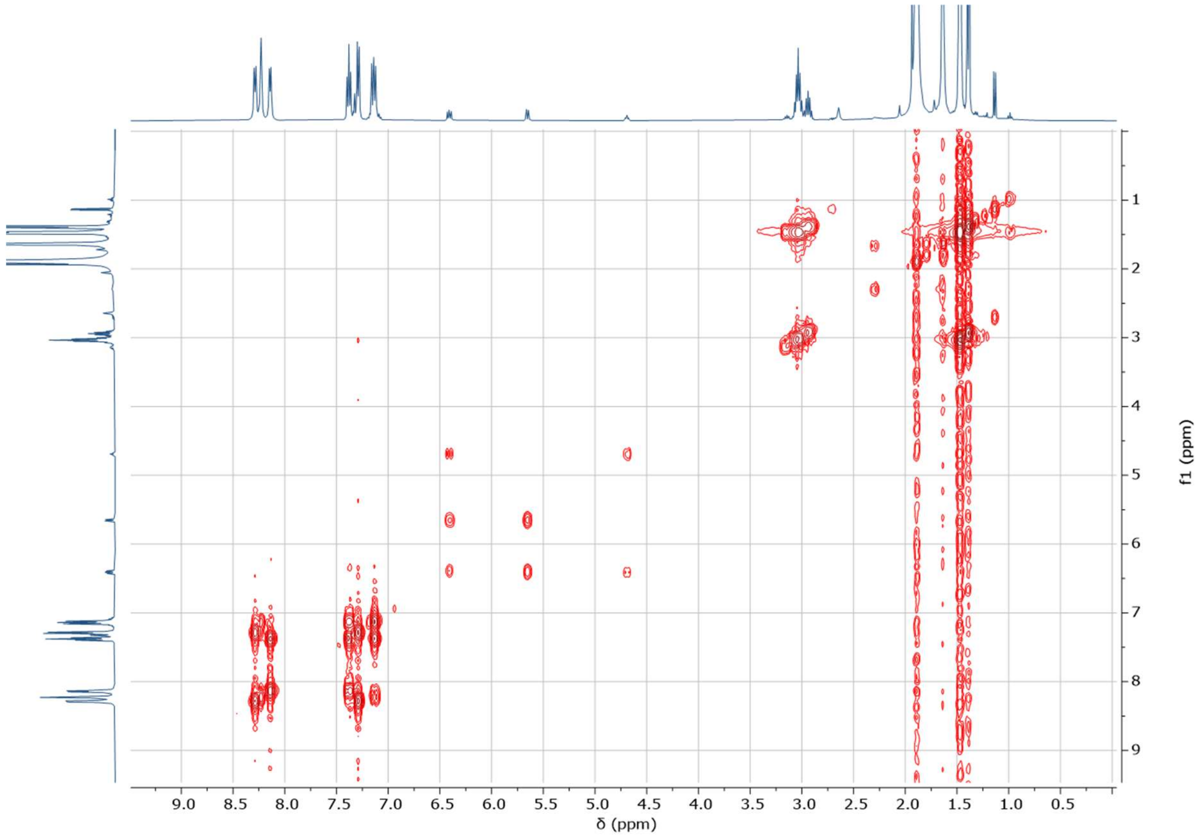

Figure S5 COSY NMR spectrum (400 MHz, C_6D_6) of **1·TMEDA**

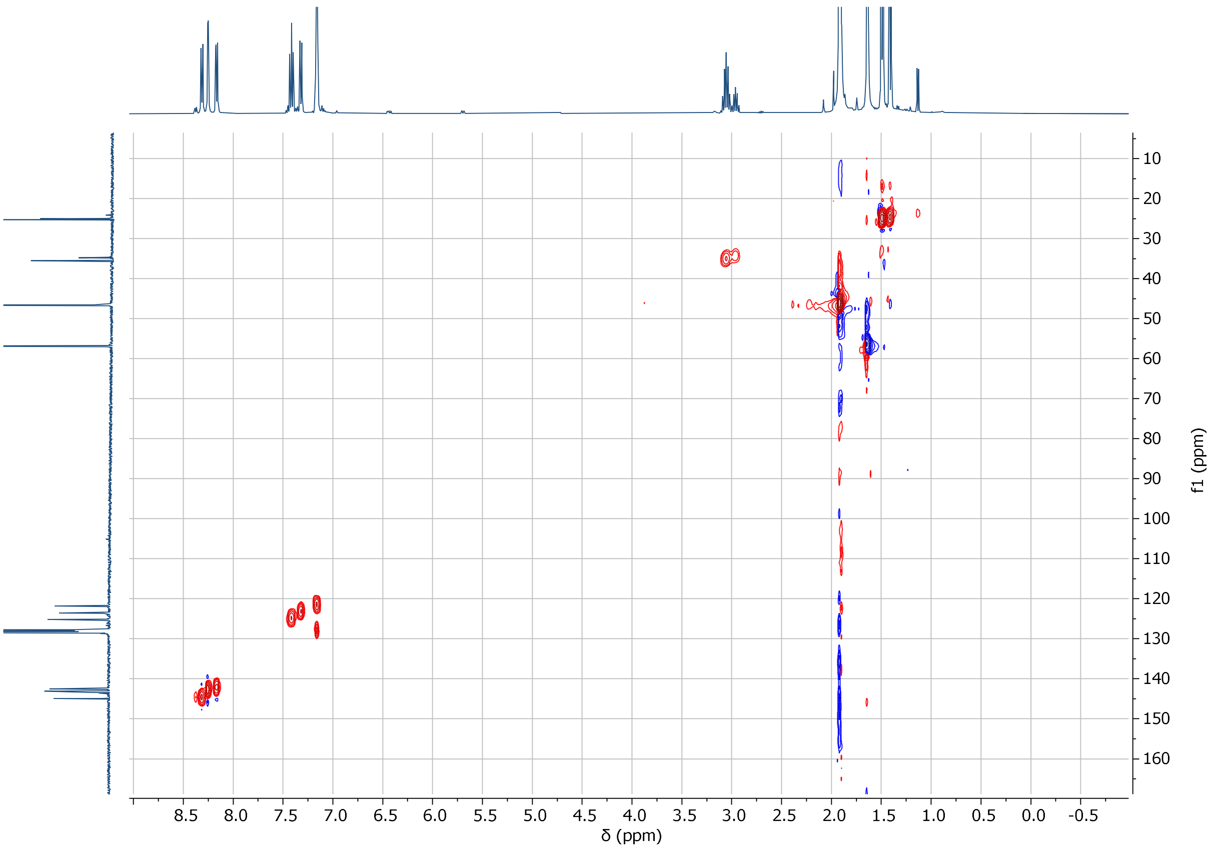

Figure S6 ^1H-^{13}C HSQC NMR spectrum of **1·TMEDA** in C_6D_6

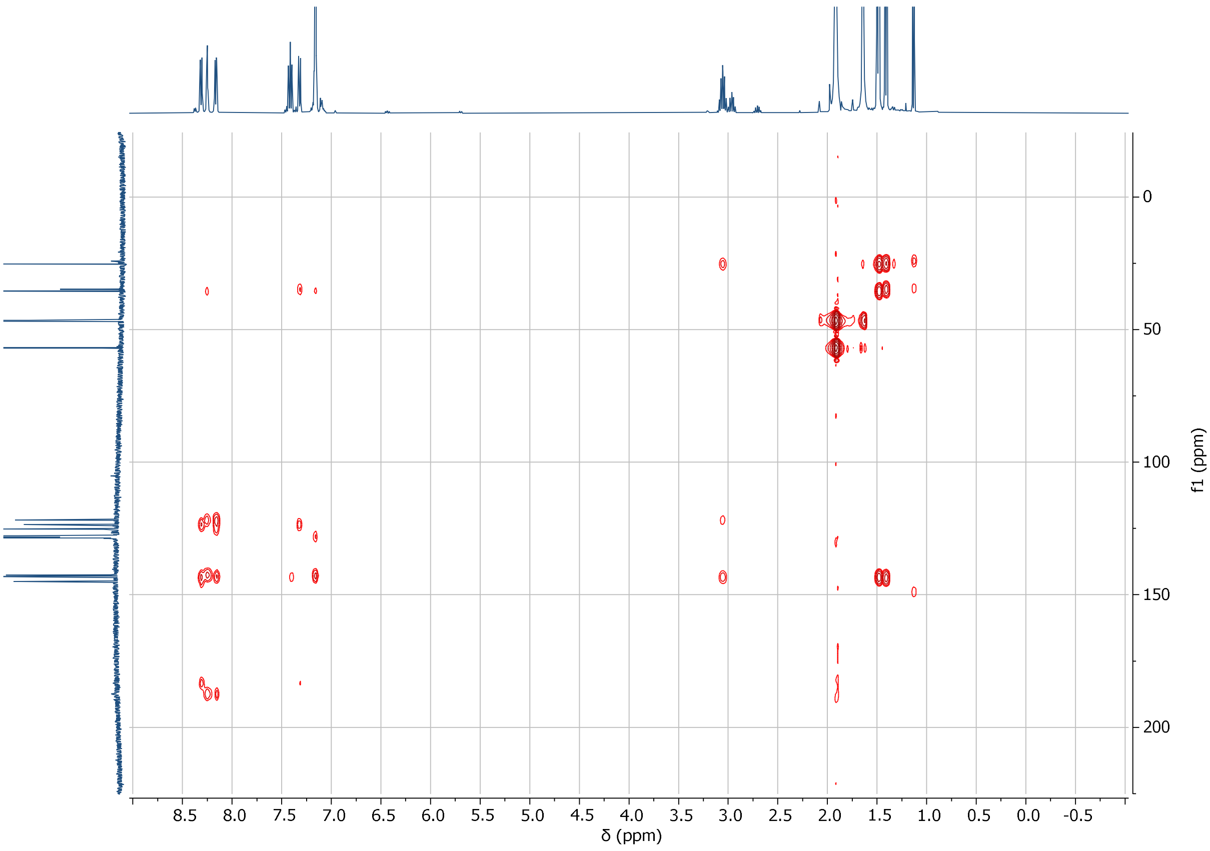

Figure S7 ^1H-^{13}C HMBC NMR spectrum of **1·TMEDA** in C_6D_6

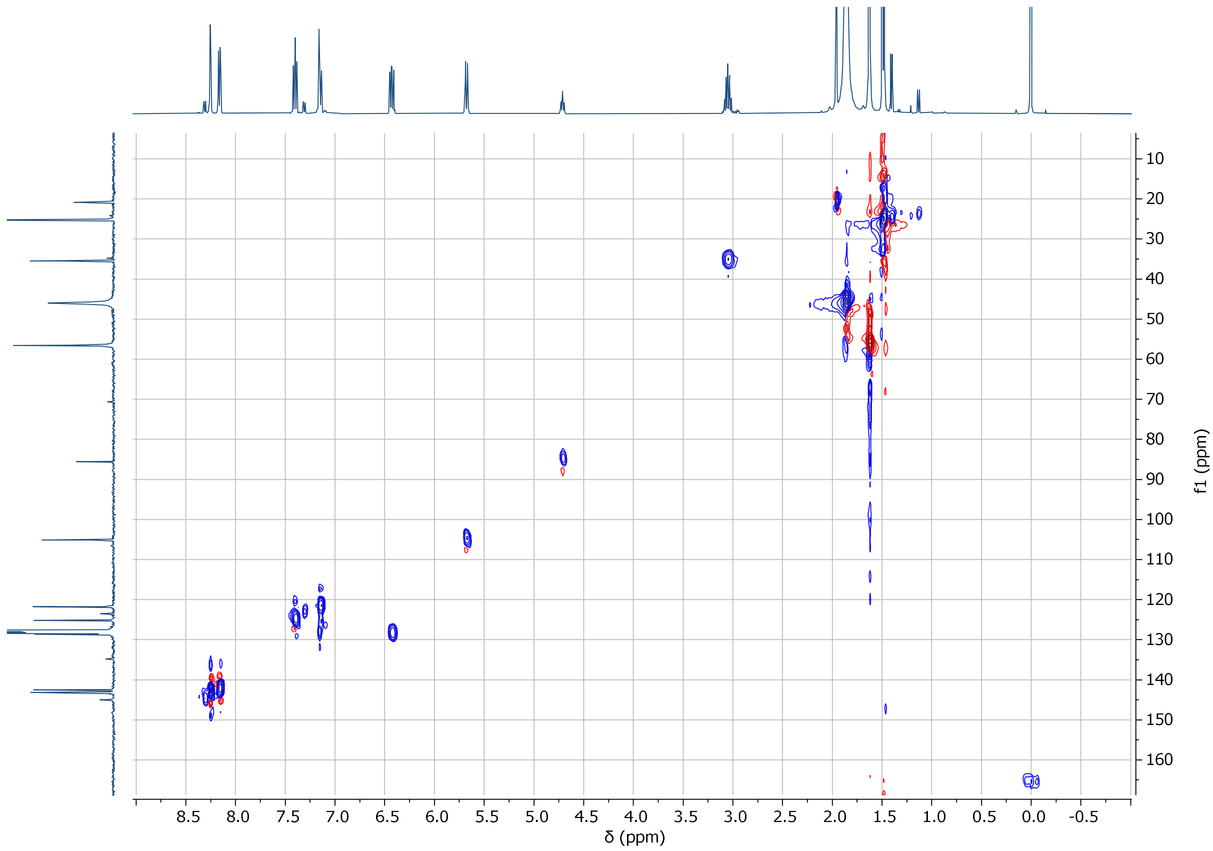

Figure S8 ^{1}H-^{13}C HSQC NMR spectrum of **1m·TMEDA** and **1α ·TMEDA** crystals in C_6D_6

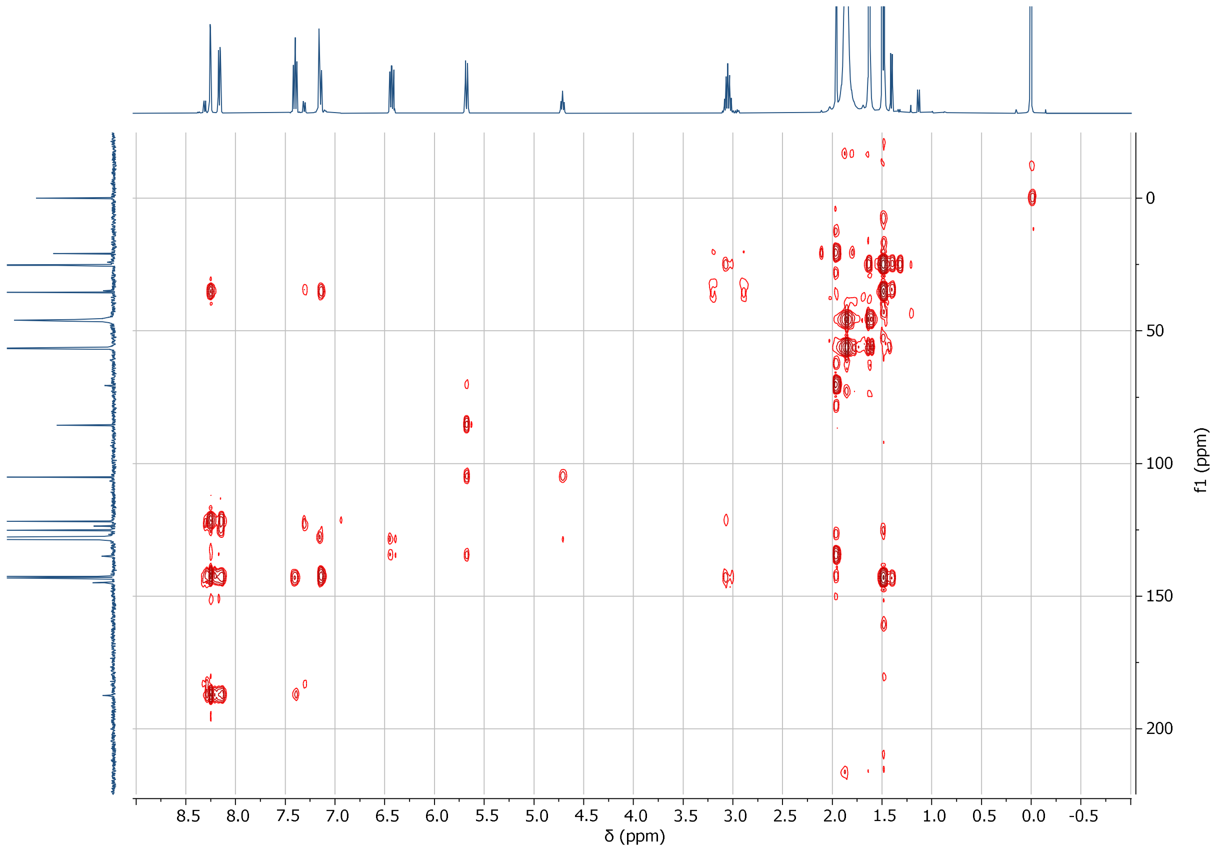

Figure S9 ^{1}H-^{13}C HMBC NMR spectrum of **1m·TMEDA** and **1α ·TMEDA** crystals in C_6D_6

2.2. *Alpha*-cumylsodium (**2·PMDETA**)

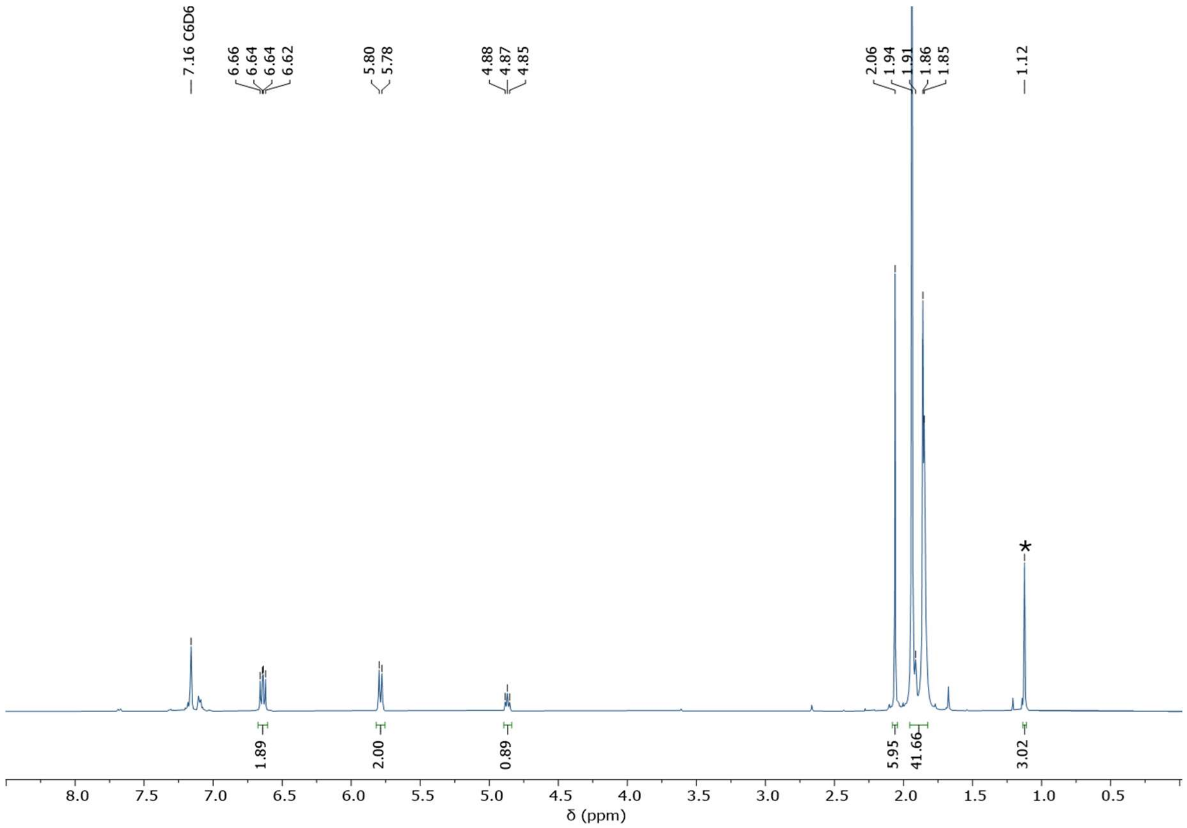

Figure S10 ^{1}H NMR spectrum (400 MHz, C_6D_6) of **2·PMDETA**, (* = free cumene)

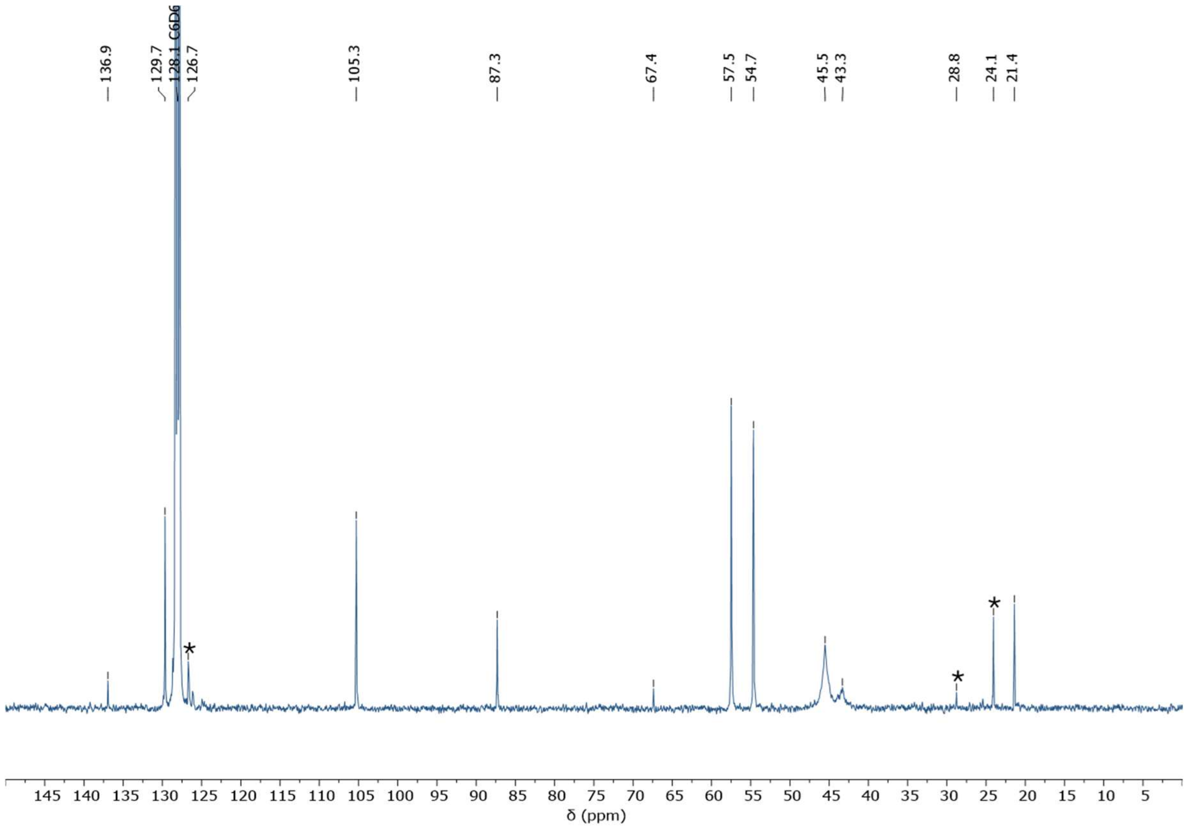

Figure S11 $^{13}C\{^{1}H\}$ NMR spectrum (101 MHz, C_6D_6) of **2·PMDETA**, (* = free cumene)

2.3. *Alpha*-cumylpotassium (**3**)

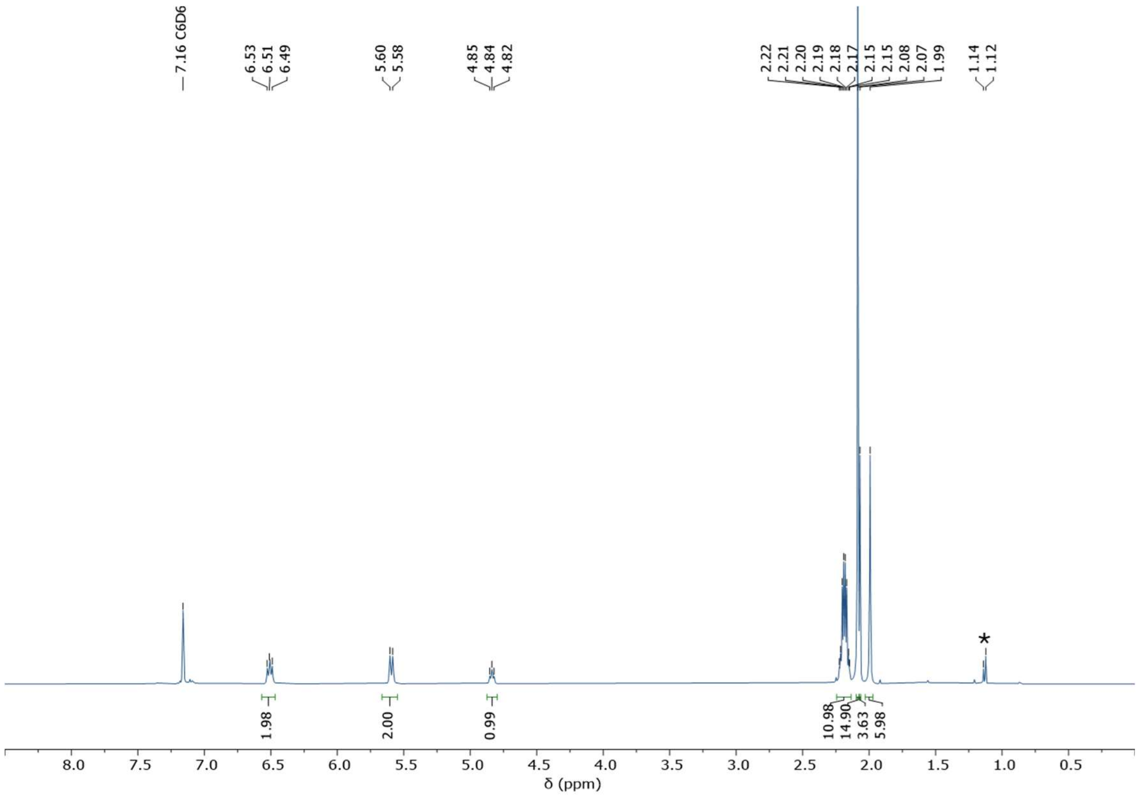

Figure S12 ^{1}H NMR spectrum (400 MHz, C_6D_6) of **3·PMDETA**, (* = free cumene)

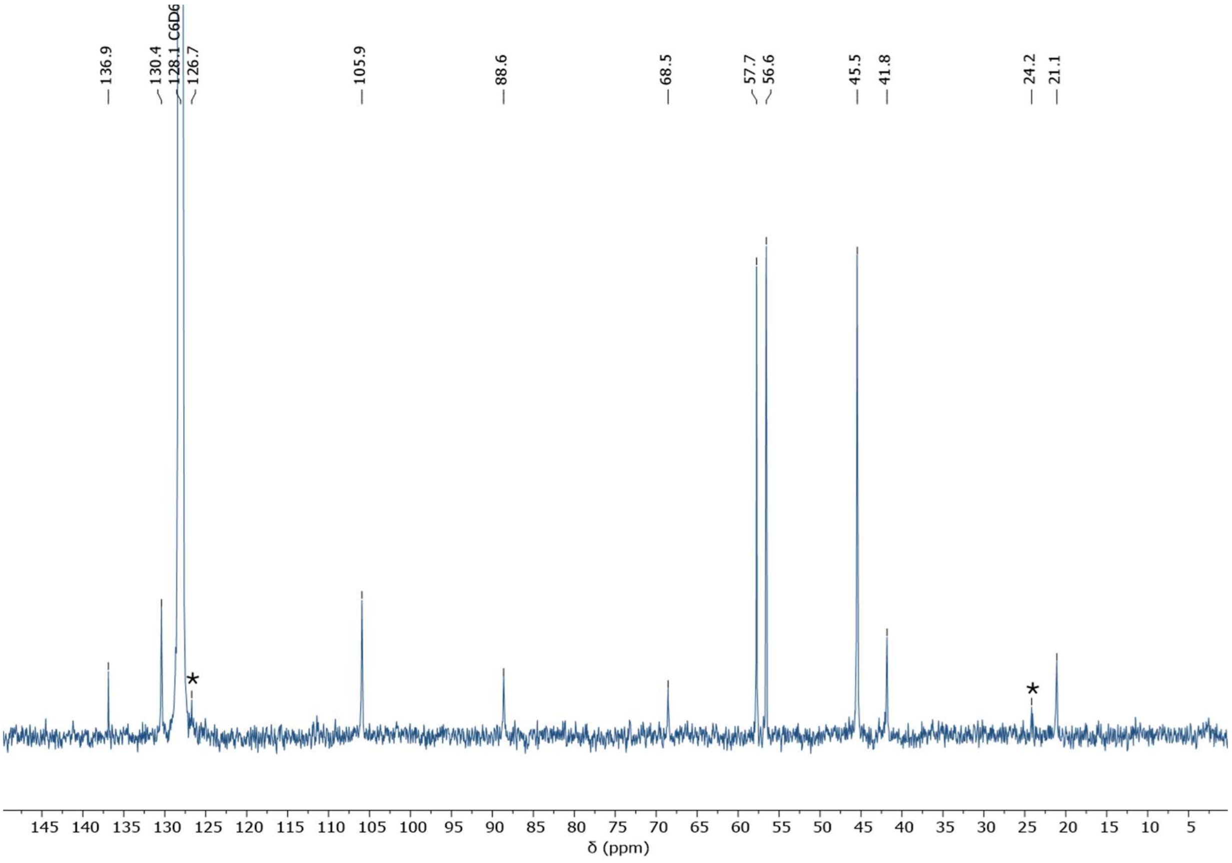

Figure S13 ^{13}C{^{1}H} NMR spectrum (101 MHz, C_6D_6) of **3·PMDETA**, (* = free cumene)

2.4. *Alpha*-cumylrubidium (**4**)

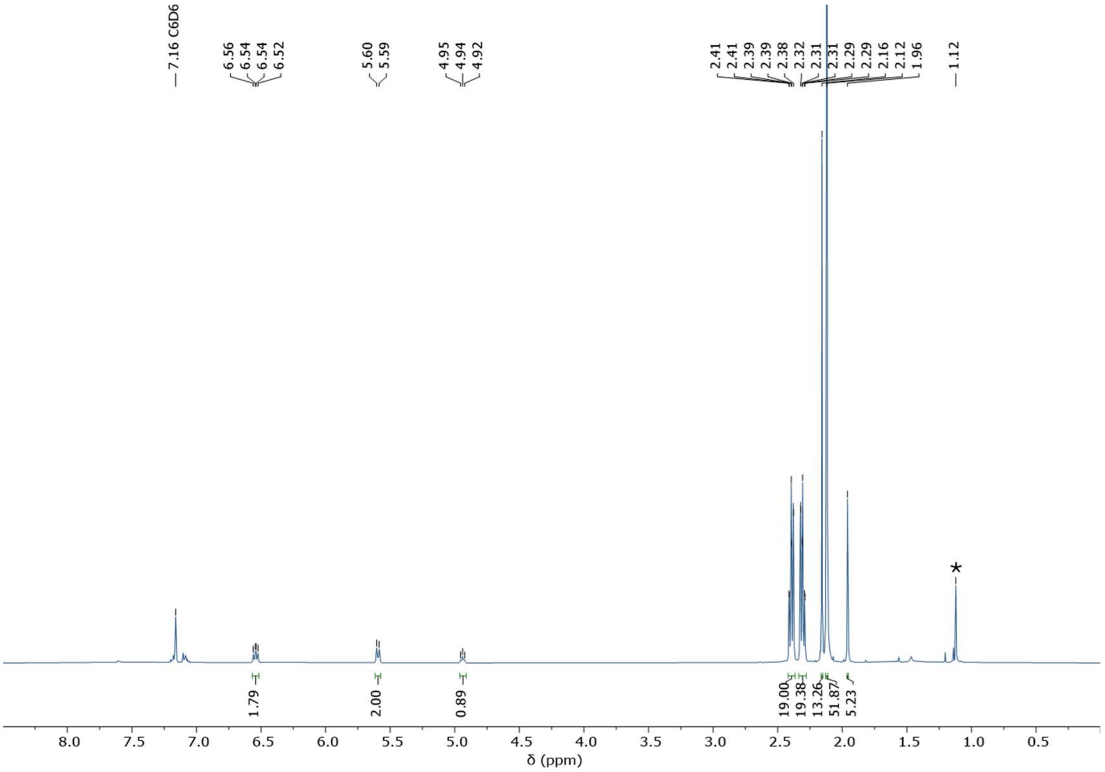

Figure S14 ^{1}H NMR spectrum (400 MHz, C_6D_6) of **4·PMDETA**, (* = free cumene)

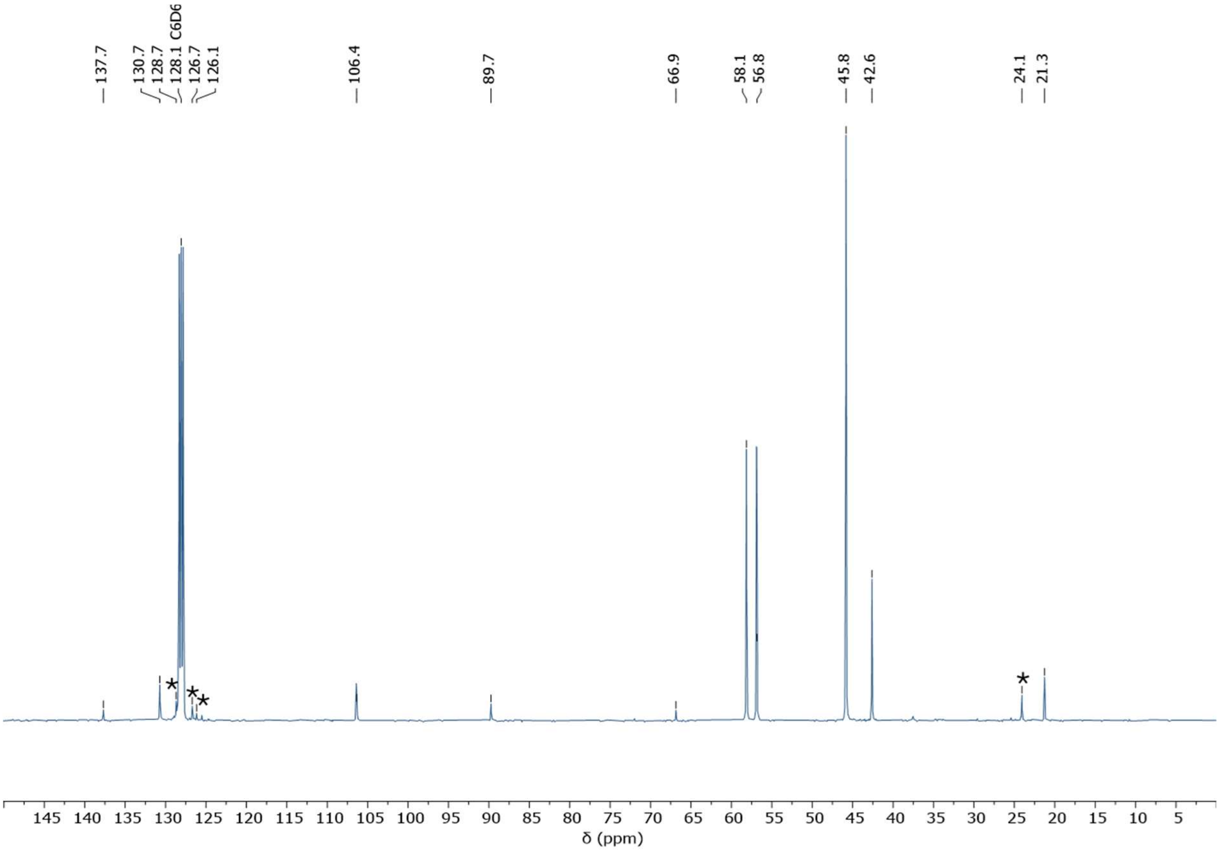

Figure S15 $^{13}C\{^{1}H\}$ NMR spectrum (101 MHz, C_6D_6) of **4·PMDETA**, (* = free cumene)

2.5. *Alpha*-cumylcesium (**5**)

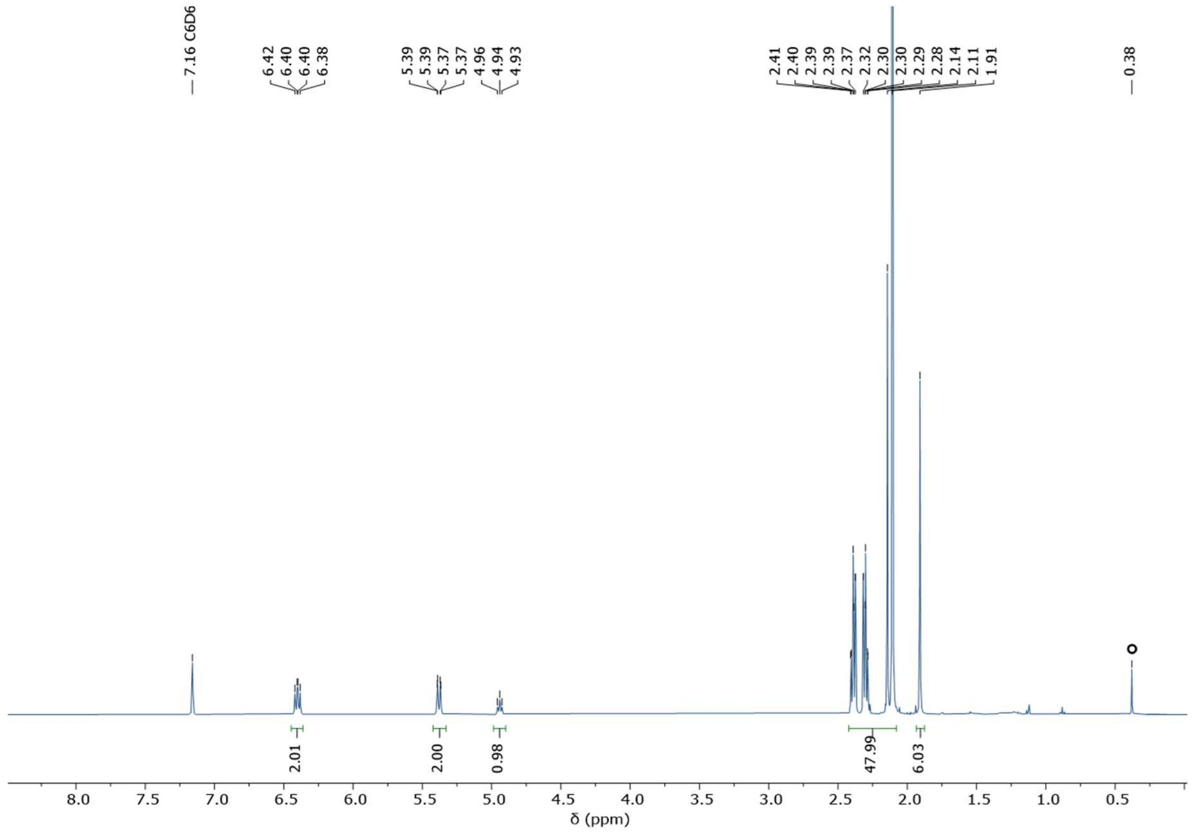

Figure S11 ^{1}H NMR spectrum (400 MHz, C_6D_6) of **5·PMDETA**, (° = impurities)

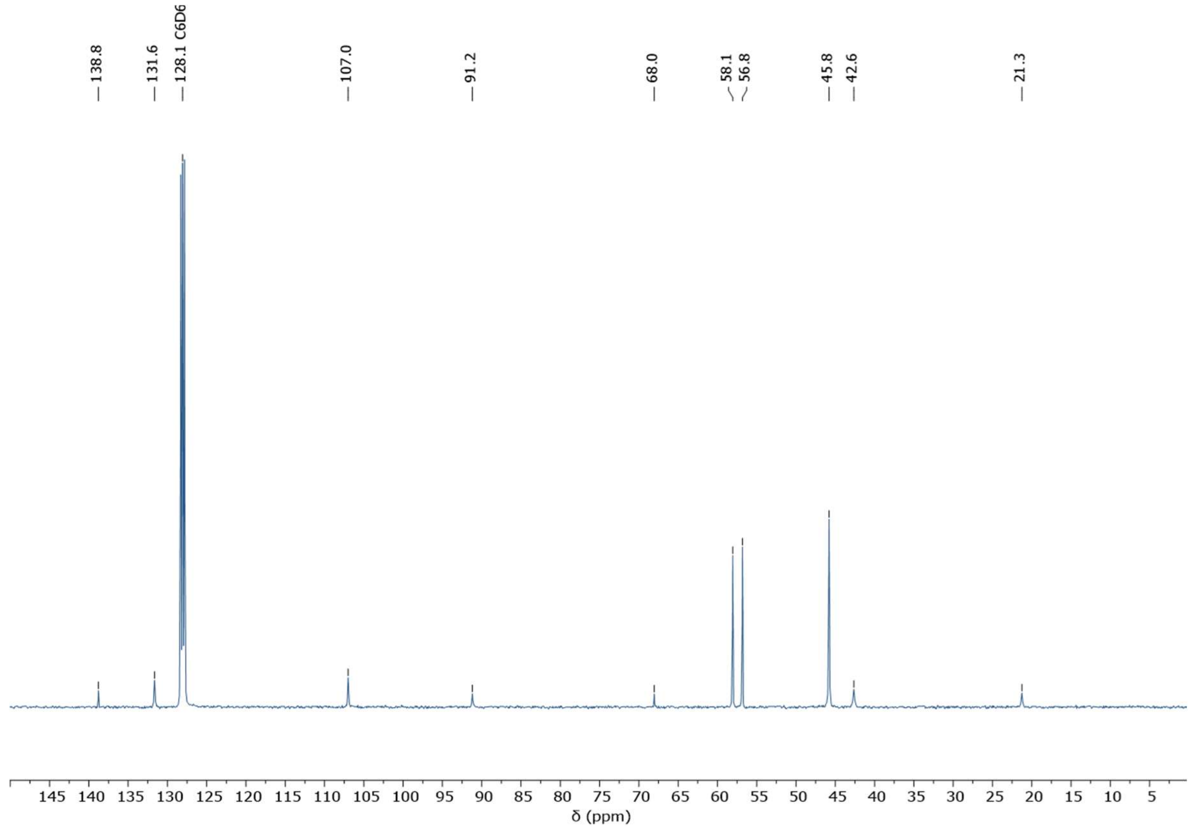

Figure S17 $^{13}C\{^{1}H\}$ NMR spectrum (101 MHz, C_6D_6) of **5·PMDETA**

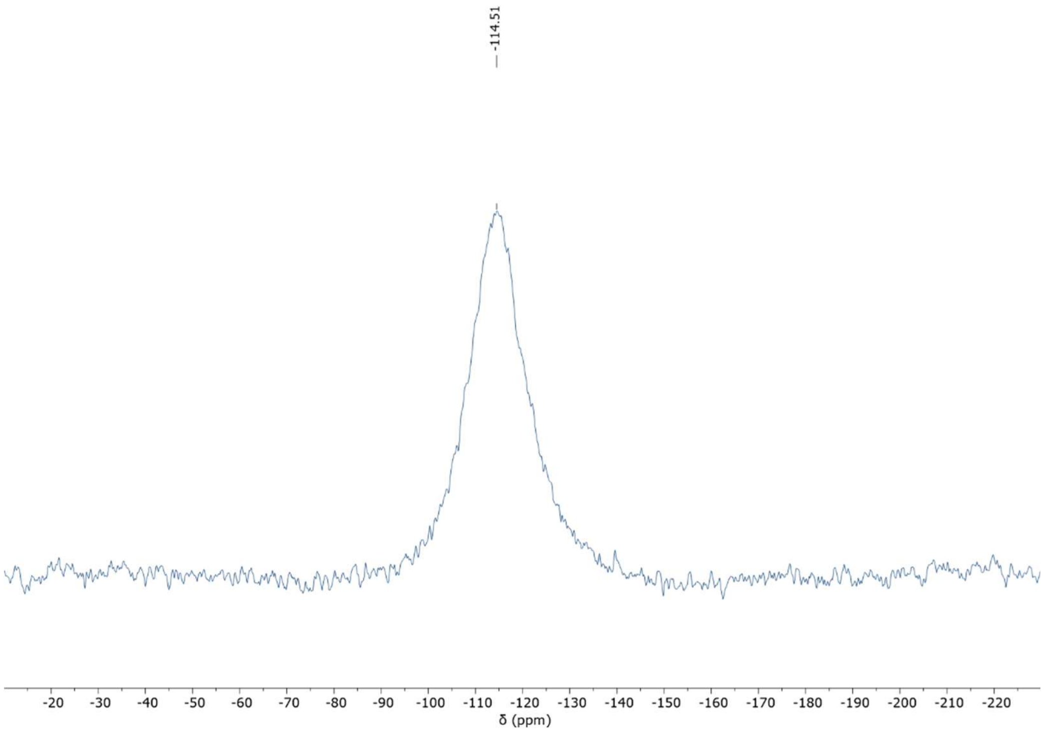

Figure S18 ^{133}Cs NMR spectrum (66 MHz, C_6D_6) of **5·PMDETA**

3. Computational Studies

All quantum chemical calculations were carried out using the Gaussian16 package.[12] The molecular structure optimisations were performed using the BP86[13,14] functional along with the 6-31G(d) basis set for HCN, def2-SVP for Rb[15] and Grimmes D3 dispersion correction.[16,17] Each stationary point was identified by a subsequent frequency calculation as minimum (Number of imaginary frequencies NIMAG: 0). Quantum Theory of Atoms in Molecules (QTAIM) topological analysis of the electron densities of **4·PMDETA** were computed with AIMAll professional (version 19.10.12)[18] using the wavefunction files obtained with Gaussian. Contour plots were generated in the AIMStudio package. Noncovalent interactions were visualized by means of the NCIPlot programme.[19]

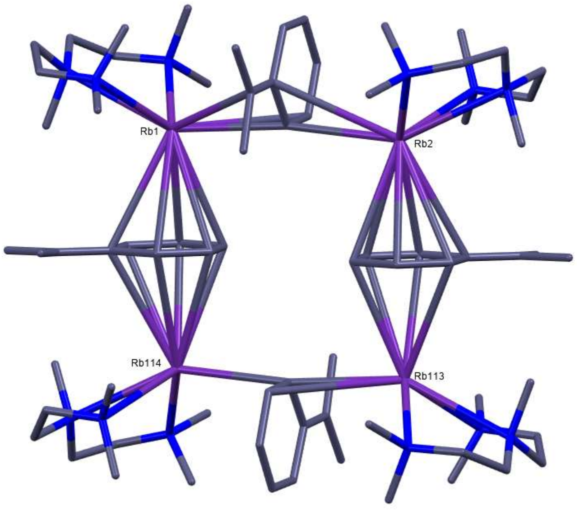

Figure S19 Optimised structure of **4·PMDETA**, highlighting the different Rb coordination modes with the cumyl ligands.

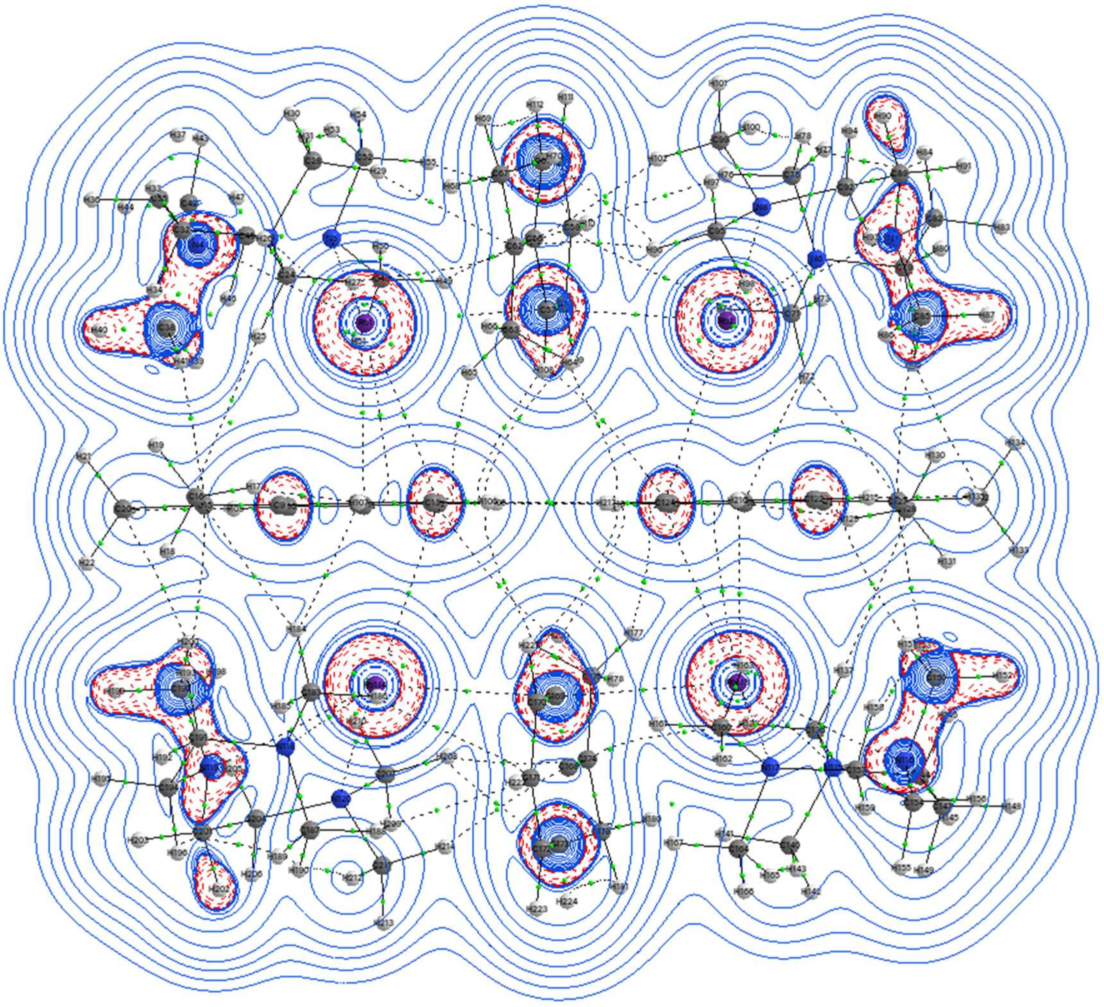

Figure S20 2D contour plot of Laplacian ($\nabla 2\ \rho$ (r)) plot across the Rb1-Rb2-Rb113 2D plane

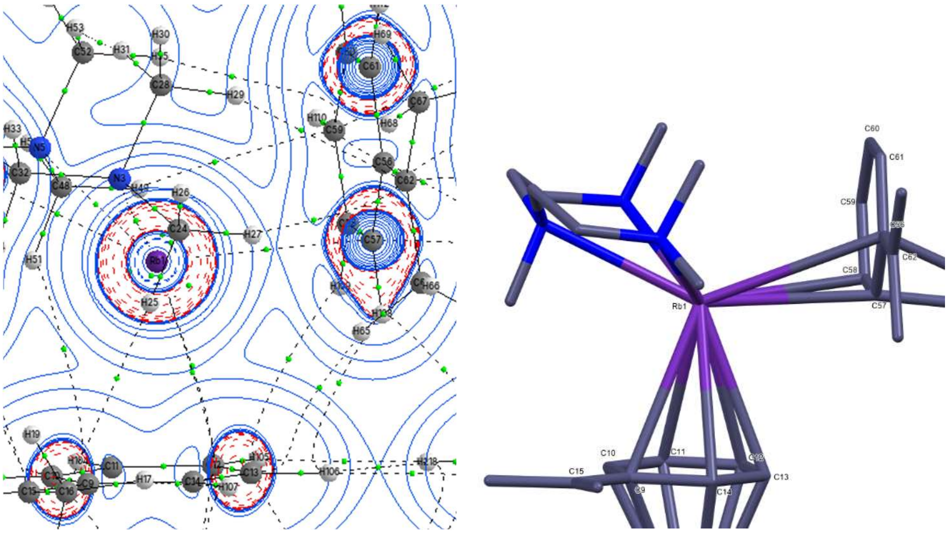

Figure S21 Zoomed in view, with DFT model to aid atom labels

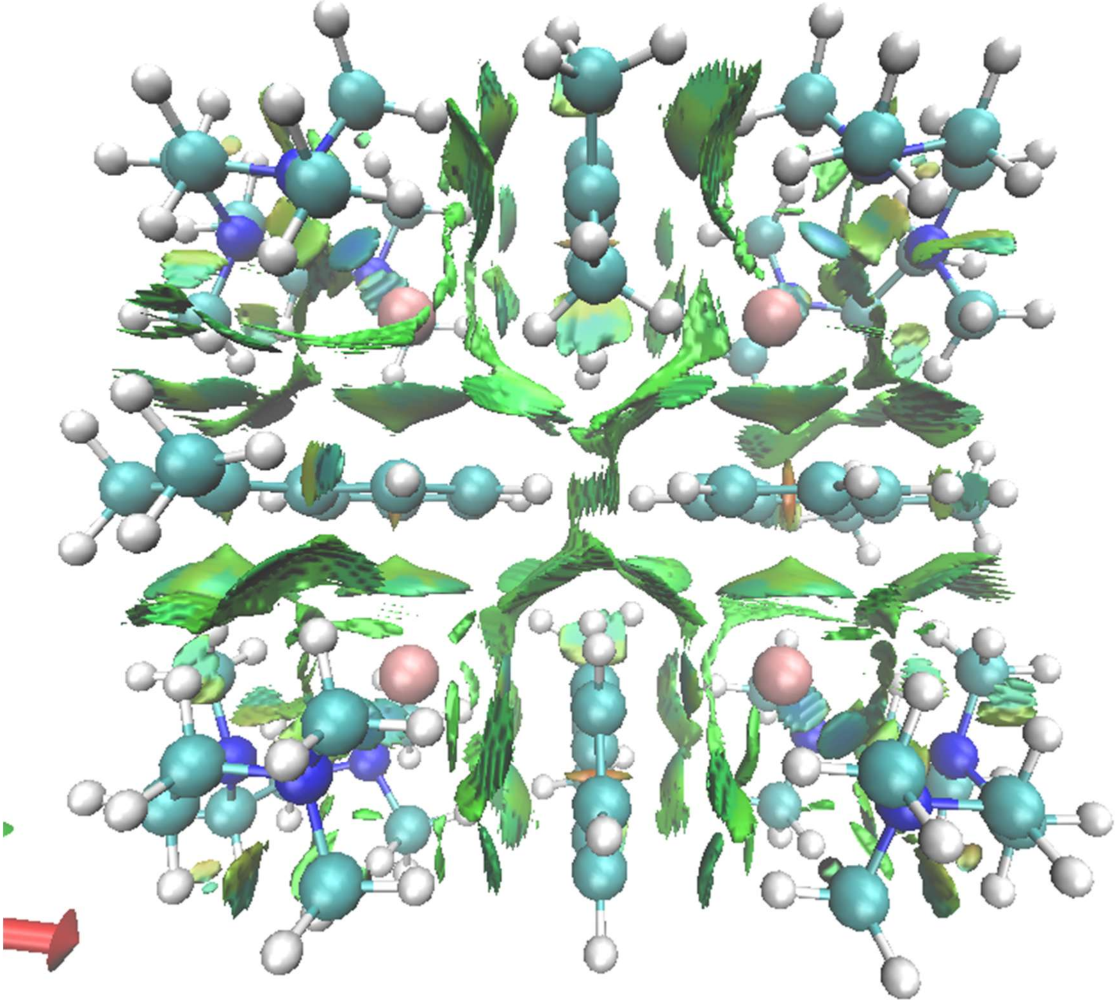

Figure S22 NCI Plot of **4·PMDETA** green areas indicate weak van der Waals interactions.

Cartesian Coordinates

Rb	-3.540600	2.469000	-0.317300
Rb	2.506400	3.409800	0.187800
N	-4.971500	3.738500	1.938800
N	-6.518500	3.327300	-0.544200
N	-4.669700	3.648200	-2.844900
N	3.451100	4.503800	-2.392400
N	5.043900	5.144700	0.083200
N	3.135300	5.453100	2.286500
C	-4.384900	-0.659200	0.659100
C	-4.455600	-0.735500	-0.807000
C	-3.343400	-0.575300	-1.635600
C	-2.041100	-0.336900	-1.122700
C	-1.922600	-0.266400	0.290900
C	-3.018200	-0.412700	1.140600
C	-5.494300	-0.798800	1.505300
C	-5.424300	-0.613000	3.002600

H	-4.434500	-0.287300	3.364900
H	-5.685700	-1.534900	3.571500
H	-6.157000	0.152800	3.347600
C	-6.898700	-1.080500	1.024500
H	-7.616900	-0.297600	1.360600
H	-7.303900	-2.034700	1.434000
H	-6.992800	-1.144400	-0.072900
C	-4.407000	3.230800	3.192000
H	-4.712500	2.180600	3.336700
H	-4.734200	3.821000	4.082900
H	-3.306800	3.276200	3.129800
C	-4.479000	5.090900	1.663700
H	-3.376300	5.069200	1.674000
H	-4.831000	5.840900	2.413500
H	-4.804300	5.420500	0.662300
C	-6.433700	3.653900	1.946900
H	-6.881500	4.311300	2.735700
H	-6.704000	2.615900	2.213900
C	-7.082200	4.039600	0.607900
H	-8.184200	3.869300	0.704400
H	-6.947400	5.122100	0.431000
C	-6.829600	1.898300	-0.484400
H	-6.398300	1.376700	-1.353800
H	-7.929700	1.702700	-0.469600
H	-6.379300	1.437200	0.410600
C	-6.950200	3.922700	-1.812900
H	-6.877100	5.020400	-1.710000
H	-8.023300	3.699200	-2.038400
C	-6.114700	3.468300	-3.021100
H	-6.294000	2.396200	-3.223600
H	-6.491700	4.024800	-3.916900
C	-3.927900	3.053000	-3.960000
H	-2.845900	3.167400	-3.772900
H	-4.168300	3.525600	-4.943800
H	-4.160300	1.975100	-4.027200
C	-4.300200	5.057200	-2.677900
H	-4.752600	5.463400	-1.757600
H	-4.625000	5.690600	-3.539900
H	-3.204800	5.131300	-2.572500
C	-0.791200	4.304200	0.723100
C	-0.528900	3.136900	-0.124700
C	-0.464600	3.209800	-1.519700
C	-0.637900	4.429200	-2.215500
C	-0.870100	5.590900	-1.424400
C	-0.944200	5.546500	-0.035800
C	-0.876100	4.204300	2.121100
C	-0.644300	2.863600	2.791000
H	0.396300	2.471700	2.691700
H	-1.289300	2.048600	2.389100
H	-0.850300	2.924100	3.876200
C	-1.138400	5.391900	3.021000
H	-1.796800	5.115100	3.870600
H	-1.643100	6.223600	2.494300
H	-0.228600	5.839800	3.486900
C	3.012200	3.503900	-3.370000
H	3.446800	2.525600	-3.108900
H	3.317500	3.758900	-4.414700

H	1.912100	3.423700	-3.334800
C	2.812100	5.795000	-2.655000
H	1.718800	5.649100	-2.681100
H	3.141900	6.250800	-3.621500
H	3.032700	6.507100	-1.841900
C	4.917200	4.577700	-2.361400
H	5.328800	4.927100	-3.342800
H	5.289600	3.547500	-2.205700
C	5.469500	5.511800	-1.273500
H	6.583600	5.527000	-1.366400
H	5.129500	6.544800	-1.467200
C	5.770700	3.966000	0.556400
H	5.373600	3.634300	1.529700
H	6.866600	4.156600	0.668000
H	5.634400	3.133300	-0.151300
C	5.149000	6.267700	1.018900
H	4.661600	7.140700	0.547800
H	6.212300	6.561900	1.209700
C	4.496400	5.987800	2.383500
H	5.102500	5.253000	2.944600
H	4.527900	6.938000	2.974500
C	2.642300	5.008400	3.592300
H	1.642900	4.559400	3.464500
H	2.560700	5.839700	4.334300
H	3.323000	4.242200	4.005000
C	2.201100	6.407600	1.680900
H	2.509900	6.641100	0.647700
H	2.132700	7.364400	2.253200
H	1.198500	5.950700	1.636500
H	-5.424800	-0.922800	-1.280600
H	-3.490300	-0.641700	-2.722900
H	-1.176600	-0.218300	-1.780900
H	-0.937000	-0.096800	0.742000
H	-2.836500	-0.344000	2.217700
H	-0.376900	2.162500	0.351400
H	-0.270500	2.283600	-2.078700
H	-0.570800	4.484100	-3.306800
H	-0.990900	6.563800	-1.921500
H	-1.112900	6.483500	0.507200
Rb	3.540600	-2.469000	0.317300
Rb	-2.506400	-3.409800	-0.187800
N	4.971500	-3.738500	-1.938800
N	6.518500	-3.327300	0.544200
N	4.669600	-3.648100	2.844900
N	-3.451100	-4.503800	2.392400
N	-5.043900	-5.144600	-0.083200
N	-3.135300	-5.453100	-2.286500
C	4.384900	0.659200	-0.659100
C	4.455600	0.735500	0.807000
C	3.343400	0.575300	1.635600
C	2.041100	0.336900	1.122700
C	1.922600	0.266400	-0.290900
C	3.018200	0.412700	-1.140600
C	5.494300	0.798800	-1.505300
C	5.424300	0.613000	-3.002600
H	4.434500	0.287300	-3.364900
H	5.685700	1.534900	-3.571400

H	6.157000	-0.152800	-3.347600
C	6.898700	1.080500	-1.024500
H	7.616900	0.297600	-1.360600
H	7.303900	2.034700	-1.434000
H	6.992800	1.144400	0.072900
C	4.407000	-3.230800	-3.192000
H	4.712500	-2.180600	-3.336700
H	4.734100	-3.821000	-4.082900
H	3.306700	-3.276200	-3.129800
C	4.478900	-5.090900	-1.663700
H	3.376300	-5.069200	-1.674000
H	4.831000	-5.840900	-2.413400
H	4.804300	-5.420500	-0.662300
C	6.433700	-3.654000	-1.946900
H	6.881400	-4.311400	-2.735700
H	6.704000	-2.615900	-2.213900
C	7.082100	-4.039700	-0.607900
H	8.184200	-3.869400	-0.704400
H	6.947400	-5.122100	-0.431000
C	6.829700	-1.898400	0.484300
H	6.398300	-1.376700	1.353800
H	7.929700	-1.702700	0.469600
H	6.379300	-1.437200	-0.410600
C	6.950200	-3.922700	1.812900
H	6.877000	-5.020400	1.710000
H	8.023300	-3.699200	2.038400
C	6.114700	-3.468300	3.021200
H	6.294000	-2.396200	3.223600
H	6.491700	-4.024700	3.917000
C	3.927900	-3.053000	3.960000
H	2.845900	-3.167300	3.772900
H	4.168300	-3.525500	4.943900
H	4.160300	-1.975000	4.027200
C	4.300200	-5.057200	2.677900
H	4.752500	-5.463400	1.757600
H	4.625000	-5.690600	3.540000
H	3.204800	-5.131300	2.572500
C	0.791200	-4.304200	-0.723100
C	0.528900	-3.136900	0.124700
C	0.464600	-3.209800	1.519700
C	0.637900	-4.429200	2.215500
C	0.870100	-5.590900	1.424400
C	0.944200	-5.546500	0.035800
C	0.876100	-4.204300	-2.121100
C	0.644300	-2.863600	-2.791000
H	-0.396300	-2.471700	-2.691700
H	1.289300	-2.048600	-2.389100
H	0.850300	-2.924100	-3.876200
C	1.138400	-5.391900	-3.021100
H	1.796800	-5.115100	-3.870600
H	1.643100	-6.223600	-2.494300
H	0.228600	-5.839800	-3.486900
C	-3.012200	-3.503900	3.370000
H	-3.446700	-2.525600	3.108900
H	-3.317400	-3.758900	4.414700
H	-1.912100	-3.423700	3.334800
C	-2.812100	-5.795000	2.655000

H	-1.718700	-5.649100	2.681100
H	-3.141900	-6.250800	3.621500
H	-3.032700	-6.507100	1.841900
C	-4.917200	-4.577700	2.361400
H	-5.328700	-4.927100	3.342800
H	-5.289600	-3.547500	2.205700
C	-5.469500	-5.511800	1.273500
H	-6.583600	-5.527000	1.366500
H	-5.129500	-6.544800	1.467200
C	-5.770700	-3.966000	-0.556400
H	-5.373600	-3.634300	-1.529700
H	-6.866600	-4.156600	-0.667900
H	-5.634400	-3.133300	0.151300
C	-5.149000	-6.267700	-1.018900
H	-4.661600	-7.140700	-0.547900
H	-6.212300	-6.561900	-1.209700
C	-4.496400	-5.987700	-2.383500
H	-5.102500	-5.253000	-2.944600
H	-4.528000	-6.938000	-2.974500
C	-2.642300	-5.008400	-3.592300
H	-1.642900	-4.559400	-3.464500
H	-2.560700	-5.839700	-4.334300
H	-3.323100	-4.242200	-4.005000
C	-2.201100	-6.407600	-1.680900
H	-2.509900	-6.641100	-0.647700
H	-2.132700	-7.364400	-2.253200
H	-1.198600	-5.950700	-1.636500
H	5.424800	0.922800	1.280600
H	3.490300	0.641700	2.722900
H	1.176600	0.218300	1.780800
H	0.937000	0.096800	-0.742100
H	2.836500	0.344000	-2.217700
H	0.376900	-2.162500	-0.351400
H	0.270500	-2.283600	2.078700
H	0.570800	-4.484100	3.306800
H	0.990900	-6.563800	1.921500
H	1.112900	-6.483500	-0.507300

4. X-ray Crystallographic Data

Crystallographic data were measured with Rigaku diffractometers using monochromated Cu-Kα (λ 1.54184 Å) radiation. Crystals were immersed in inert oil prior to mounting on the X-ray diffractometer. All data was processed with CrysAlisPro [20] software. Structures were solved using the ShelXT [21] program, and refined to convergence against F^2 and all unique reflections with ShelXL-2018,[22] as implemented within the software Olex2 or WinGX.[23,24]

The structure of **1m·TMEDA** is of lower quality than is desirable. All samples measured gave streaked diffraction rather than diffraction spots and all were clearly not single crystals. The best model obtained treated the measured reflections as twinned (matrix used 0.0801 -0.0205 -0.4626 -0.0156 -0.9993 0.0069 -2.1477 0.0380 -0.0802) and utilised refinement against a hklf 5 formatted reflection file. The relative sizes of the twin domains refined to 0.799(3):0.2001(3). The TMEDA groups were modelled as disordered over two sites with the occupancy of the major site refined to 0.552(7). Appropriate restraints and constraints were utilised to ensure that this large disordered fragment approximated to normal geometry and normal displacement behaviour. Attempts to find a non-disordered solution (e.g. a larger unit cell, lower symmetry models) were fruitless. The structure is included herein as we believe that, taken with the other characterisation discussed, it shows the chemical identity of the species. For this structure, no reliance or importance is placed in the paper on structural detail (e.g. bond lengths, bond angles).

Three crystallographically independent formula units of the complex are present in the asymmetric unit for **5·PMDETA**. One of the three independent cumyl anions was modelled as disordered by a rotation of 180°. Occupancy of the major site refined to 0.681(12). Again, Appropriate restraints were utilised to ensure that this disordered fragment approximated to normal geometry and normal displacement behaviour.

Selected crystallographic and refinement data are given in Table S2. Full crystallographic details in cif format have been deposited with the CCDC, see deposition numbers 2515570 to 2515575.

Table S3 Selected crystallographic data and structure refinement details

Complex	**1m ·TMEDA**	**1α ·TMEDA**	**2·PMDETA**	**3·PMDETA**	**4·PMDETA**	**5·PMDETA**
CCDC	2515570	2515571	2515572	2515573	2515574	2515575
Empirical formula	$C_{30}H_{54}Li_2N_4$	$C_{15}H_{27}LiN_2$	$C_{18}H_{34}N_3Na$	$C_{18}H_{34}KN_3$	$C_{72}H_{136}N_{12}Rb_4$	$C_{18}H_{34}N_3Cs$
Formula weight	484.65	242.32	315.47	331.58	1511.80	425.39
Temperature/K	100(2)	100(2)	173(2)	150(2)	100(2)	150(2)
Crystal system	monoclinic	monoclinic	orthorhombic	monoclinic	monoclinic	orthorhombic
Space group	P2/c	$P2_1/n$	Pbca	$P2_1$	$P2_1/c$	$P\ 2_1\ 2_1\ 2_1$
a/Å	9.2775(11)	11.4777(1)	16.76942(8)	9.7724(1)	14.6546(1)	11.9428(1)
b/Å	8.7939(10)	11.4199(2)	13.03331(6)	10.9866(1)	14.9842(1)	18.3415(2)
c/Å	19.779(2)	11.7698(1)	17.78221(9)	9.8878(1)	19.7335(2)	29.1229(3)
α/°	90	90	90	90	90	90
β/°	98.657(10)	92.627(1)	90	109.386(1)	110.437(1)	90
γ/°	90	90	90	90	90	90
U/Å^3	1595.3(3)	1541.10(3)	3886.50(3)	1001.418(18)	4060.48(6)	6379.34(11)
Z	2	4	8	2	2	12
μ/mm^{-1}	0.431	0.446	0.679	2.309	3.341	13.574
2Θ range for data collection/°	9.046 - 137.990	10.528 - 143.714	9.930 - 148.968	9.482 - 146.042	6.436 - 146.730	5.694 - 143.996
Reflections collected	14848*	11455	38782	13593	55619	38777
Independent reflections	7580	2995	3969	3867	8128	11209
R_{int}	0.0378*	0.0214	0.0189	0.0233	0.0365	0.0561
Reflections observed	5081	2719	3754	3828	7760	10350
Parameters	199	169	336	206	452	677
Goodness-of-fit on F^2	1.618	1.050	1.038	1.051	1.069	1.049
Final R1, wR2 [I>=2σ (I)]	0.1266, 0.3898	0.0451, 0.1271	0.0293, 0.0842	0.0216, 0.0588	0.0221, 0.0540	0.0383, 0.0933
Final R1, wR2 [all data]	0.15610, 0.4196	0.0482, 0.1300	0.0308, 0.0854	0.0218, 0.0590	0.0236, 0.0548	0.0419, 0.0952
Largest diff. peak/hole / e Å^{-3}	0.629/-0.249	0.271/-0.199	0.213/-0.166	0.204/-0.171	0.324/-0.532	1.321/-0.588

* Treated as a twin. These values were for the data prior to generation of a hklf 5 formatted reflection file.

5. DOSY Spectroscopy

2D 1H Diffusion-Ordered Spectroscopy (DOSY) spectra were recorded on a Bruker AV400 spectrometer operating at 400.1 MHz for 1H and measured at 300 K. An estimate of the molecular weight (MW) of the species in solution was obtained via comparison of the diffusion coefficients of $\mathbf{1}^{\mathbf{AM}}$·(donor) (AM = Li, donor = TMEDA and AM = Na-Cs, donor = PMDETA) and either an internal standard of TMS or the residual C_6D_6 solvent signal to external calibration curves (ECCs) with normalised diffusion coefficients.[25-27]

5.1. DOSY of **1m·TMEDA** and **1α·TMEDA**

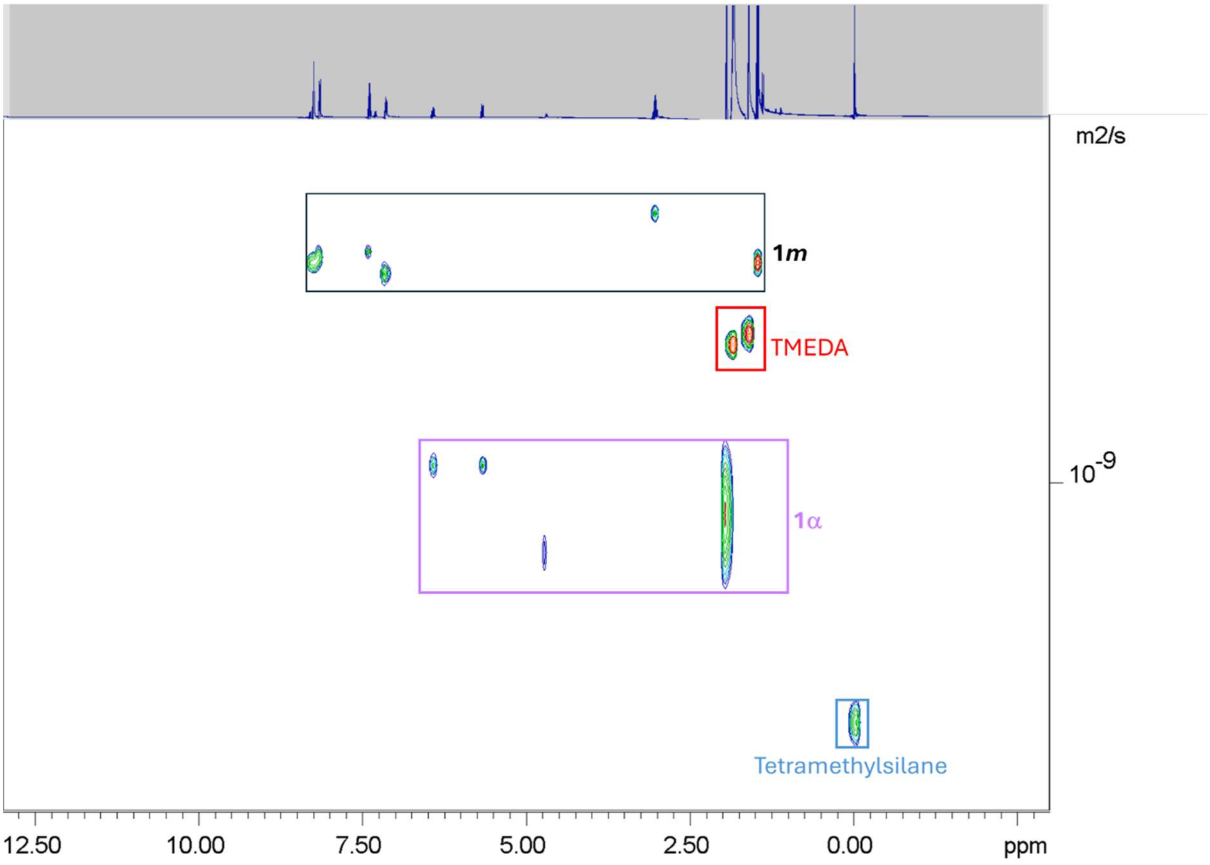

Figure S23 ^{1}H DOSY NMR spectrum of **1m·TMEDA and 1α ·TMEDA** in C_6D_6

Table S4 Diffusion coefficients and corresponding data parameters from 2D ^{1}H DOSY NMR spectrum of **1m·TMEDA** and **1α·TMEDA** in C_6D_6

Peak Name	*F2 (ppm)*	*D (m^2s^{-1})*	*error*	*log D*
***1 Li*-m**	8.247	6.43E-10	3.05E-12	-9.1918
***2 Li*-m**	8.157	6.52E-10	1.42E-11	-9.1858
***3 Li*-m**	7.396	6.48E-10	8.77E-12	-9.1884
***4 Li*-m**	7.141	6.67E-10	6.43E-12	-9.1759
5 Li-alpha	6.423	9.65E-10	1.49E-11	-9.0155
6 Li-alpha	5.667	9.73E-10	6.87E-12	-9.0119
7 Li-alpha	4.706	1.51E-09	1.43E-10	-8.8210
***8 Li*-m**	3.045	6.07E-10	1.28E-11	-9.2168
9 Li-alpha	1.954	1.01E-09	1.20E-10	-8.9957
10 TMEDA	1.855	7.62E-10	4.97E-13	-9.1180
11TMEDA	1.621	7.70E-10	9.98E-12	-9.1135
***12 Li*-m**	1.481	6.59E-10	2.31E-11	-9.1811
13 TMS	-0.005	1.60E-09	4.36E-11	-8.7959
Li-alpha (avg)	–	1.11E-09	–	-8.9885
Li-m (avg)	–	6.46E-10	–	-9.1898
TMEDA (avg)	–	7.66E-10	–	-9.1158

Table S5 MW and MW_{DOSY} for potential species in **1m·TMEDA and 1α·TMEDA** and the calculated deviation from their theoretical MW (MW_{diff})

Species	*MW (g mol^{-1})*	*ECC*	*MW_{DOSY} (g mol^{-1})*	*MW_{diff} (%)*
Li(C_9H_{11}) (meta)	126	DSE	431	-44
[Li(C_9H_{11})$_2$TMEDA$_2$] (meta)	484	DSE	431	12
[Li(C_9H_{11})] (alpha)	126	DSE	219	-42
[Li(C_9H_{11})TMEDA] (alpha)	242	DSE	219	11
TMEDA (avg)	116	DSE	327	-65

5.2. DOSY of **2·PMDETA** crystals

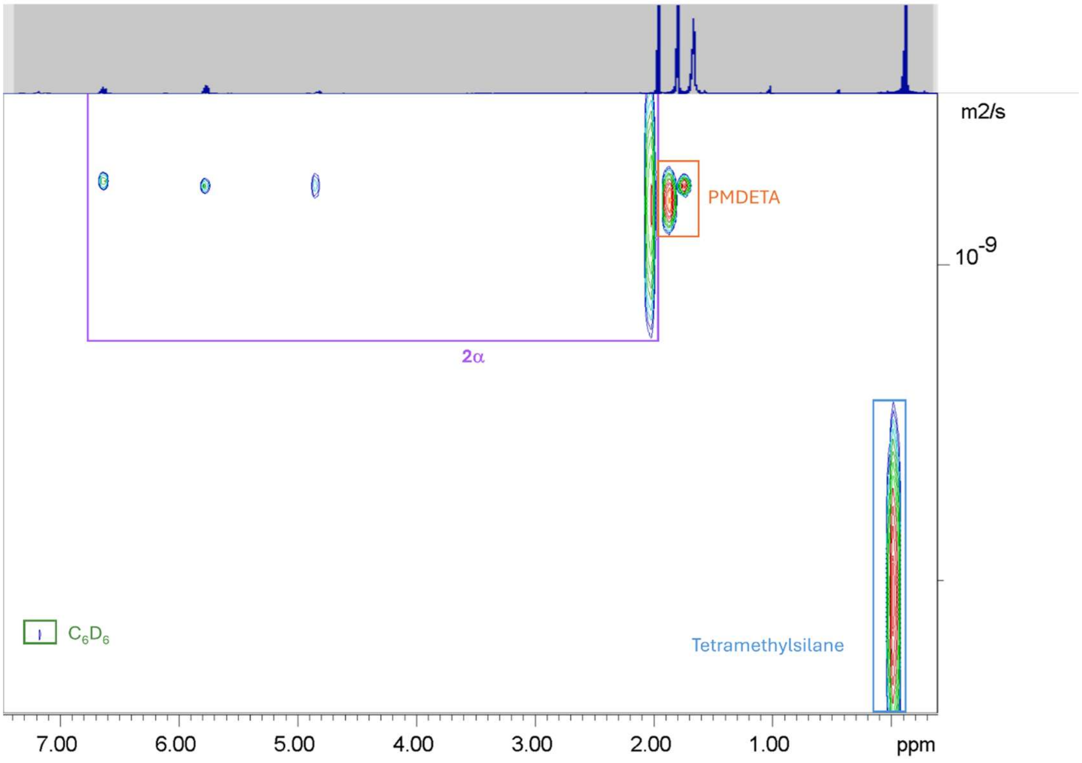

Figure S24 ^{1}H DOSY NMR spectrum of **2·PMDETA** in C_6D_6

Table S6 Diffusion coefficients and corresponding data parameters from 2D ^{1}H DOSY NMR spectrum of **2·PMDETA** in C_6D_6

Peak Name	*F2 (ppm)*	D (m^2s^{-1})	*error*	*log* D
1 C_6D_6	7.154	1.870E-09	1.082E-10	-8.7282
2 Na	6.614	8.320E-10	1.118E-11	-9.0799
3 Na	5.770	8.410E-10	9.451E-12	-9.0752
4 Na	4.843	8.390E-10	2.20E-11	-9.0762
5 Na	2.028	8.740E-10	1.398E-10	-9.0585
6 PMDETA	1.872	8.670E-10	2.818E-11	-9.0620
7 PMDETA	1.865	7.880E-10	4.049E-11	-9.1035
8 PMDETA	1.745	8.410E-10	9.795E-12	-9.0752
9 TMS	-0.002	1.810E-09	3.182E-11	-8.7423
Na (avg)	–	8.47E-10	–	-9.0724
PMDETA (avg)	–	8.32E-10	–	-9.0799

Table S7 MW and MW_{DOSY} for potential species in **2·PMDETA** and the calculated deviation from their theoretical MW (MW_{diff})

Species	*MW (g mol^{-1})*	*ECC*	*MW_{DOSY} (g mol^{-1})*	*MW_{diff} (%)*
$Na(C_9H_{11})$	142	DSE	340	-58
$[Na(C_9H_{11})(PMDETA)]$	315	DSE	340	-7
PMDETA (avg)	173	DSE	350	-51

5.3. DOSY of **3·PMDETA** crystals

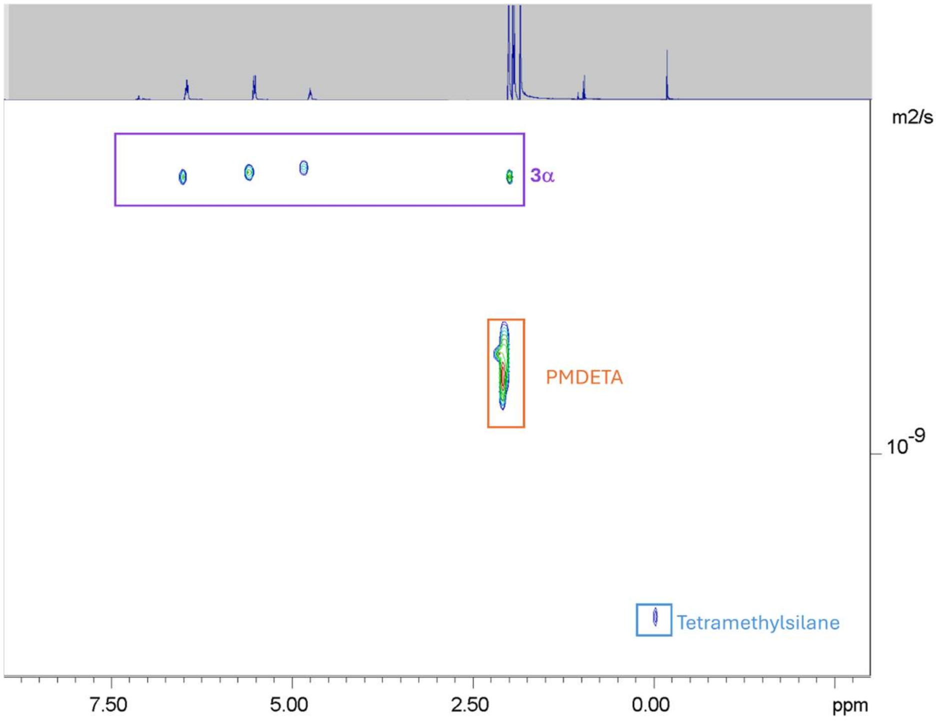

Figure S25 ^{1}H DOSY NMR spectrum of **3·PMDETA** in C_6D_6

Table S8 Diffusion coefficients and corresponding data parameters from 2D ^{1}H DOSY NMR spectrum of **3·PMDETA** in C_6D_6

Peak Name	*F2 (ppm)*	*D (m^2s^{-1})*	*error*	*log D*
1 K	6.502	3.96E-10	7.286E-12	-9.4023
2 K	5.580	4.03E-10	4.058E-12	-9.3947
3 K	4.827	3.84E-10	7.190E-12	-9.4157
4 PMDETA	2.145	7.43E-10	1.015E-11	-9.1290
5 PMDETA	2.094	7.61E-10	1.762E-11	-9.1186
6 PMDETA	2.064	7.77E-10	4.469E-12	-9.1096
7 K	1.984	4.40E-10	1.315E-11	-9.3565
8 TMS	-0.005	1.60E-09	5.028E-11	-8.7959
K (avg)	–	4.06E-10	–	-9.3917
PMDETA (avg)	–	7.60E-10	–	-9.1190

Table S9 MW and MW_{DOSY} for potential species in **3·PMDETA** and the calculated deviation from their theoretical MW (MW_{diff})

Species	*MW (g mol^{-1})*	*ECC*	*MW_{DOSY} (g mol^{-1})*	*MW_{diff} (%)*
$K(C_9H_{11})$	158	DSE	909	-83
$[K(C_9H_{11})]_6$	950	DSE	909	5
$[K(C_9H_{11})_2(PMDETA)_3]$	836	DSE	909	-8
$[K(C_9H_{11})_3(PMDETA)_3]$	995	DSE	909	9
PMDETA (avg)	173	DSE	331	-48

5.4. DOSY of **4·PMDETA** crystals

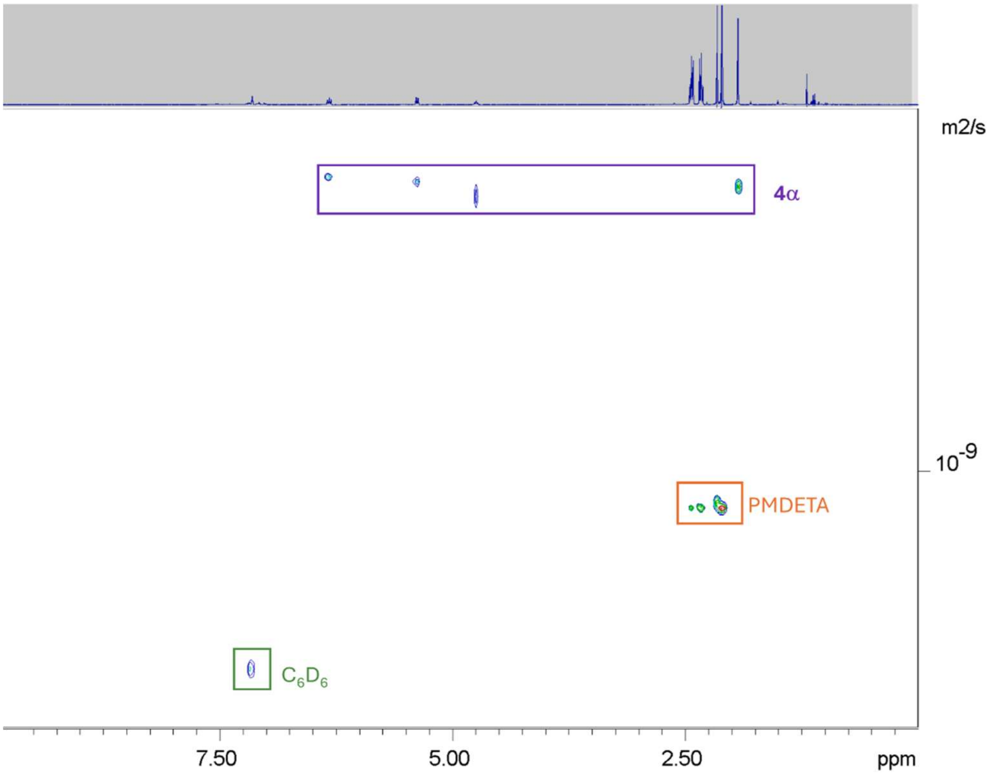

Figure S26 ^{1}H DOSY NMR spectrum of **4·PMDETA** in C_6D_6

Table S10 Diffusion coefficients and corresponding data parameters from 2D ^{1}H DOSY NMR spectrum of **4·PMDETA** in C_6D_6

Peak Name	*F2 (ppm)*	*D (m^2s^{-1})*	*error*	*log D*
1 C_6D_6	7.155	1.660E-09	7.381E-11	-8.7799
2 Rb	6.331	4.220E-10	6.243E-12	-9.3747
3 Rb	5.392	4.060E-10	7.675E-12	-9.3915
4 Rb	4.761	4.050E-10	1.380E-11	-9.3925
5 PMDETA	2.450	1.110E-09	1.198E-11	-8.9547
6 PMDETA	2.346	1.120E-09	1.159E-11	-8.9508
7 PMDETA	2.171	1.110E-09	1.673E-11	-8.9547
8 PMDETA	2.118	1.130E-09	5.641E-11	-8.9469
9 Rb	1.947	4.330E-10	1.232E-11	-9.3635
Rb (avg)	–	4.17E-10	–	-9.3804
PMDETA (avg)	–	1.12E-09	–	-8.9518

Table S11 MW and MW_{DOSY} for potential species in **4·PMDETA** and the calculated deviation from their theoretical MW (MW_{diff})

Species	*MW (g mol^{-1})*	*ECC*	*MW_{DOSY} (g mol^{-1})*	*MW_{diff} (%)*
$Rb(C_9H_{11})$	205	DSE	811	-75
$[Rb(C_9H_{11})]_4$	820	DSE	811	+1
PMDETA (avg)	173	DSE	173	0

5.5. DOSY of **5·PMDETA** crystals

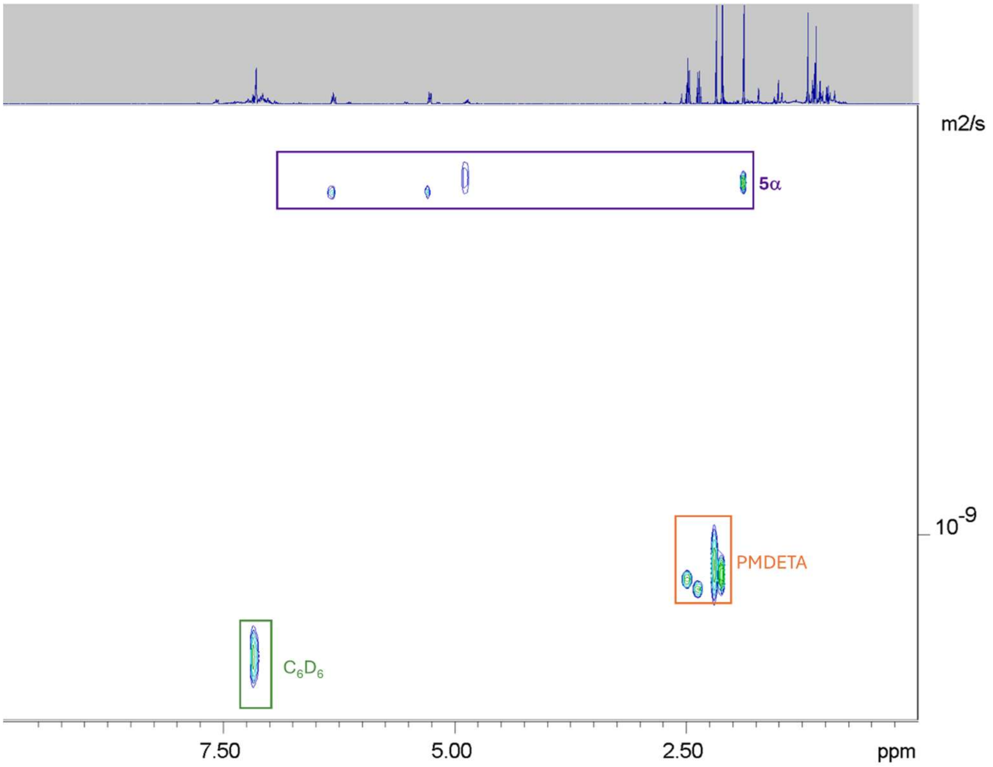

Figure S27 ^{1}H DOSY NMR spectrum of **5·PMDETA** in C_6D_6

Table S22 Diffusion coefficients and corresponding data parameters from 2D ^{1}H DOSY NMR spectrum of **5·PMDETA** in C_6D_6

Peak Name	F2 (ppm)	D (m^2s^{-1})	error	log D
1 C_6D_6	7.155	1.630E-09	4.333E-11	-8.7878
2 Cs	6.318	3.640E-10	2.908E-11	-9.4389
3 Cs	5.278	3.780E-10	2.146E-11	-9.4225
4 Cs	4.877	3.750E-10	7.686E-11	-9.4260
5 PMDETA	2.498	1.110E-09	2.612E-11	-8.9547
6 PMDETA	2.375	1.120E-09	2.849E-11	-8.9508
7 PMDETA	2.195	1.200E-09	5.986E-11	-8.9208
8 PMDETA	2.125	1.140E-09	4.418E-11	-8.9431
9 Cs	1.895	3.740E-10	1.626E-11	-9.4271
Cs (avg)	–	3.73E-10	–	-9.4286
PMDETA (avg)	–	1.14E-09	–	-8.9421

Table S13 MW and MW_{DOSY} for potential species in **5·PMDETA** and the calculated deviation from their theoretical MW (MW_{diff})

Species	MW ($g\ mol^{-1}$)	ECC	MW_{DOSY} ($g\ mol^{-1}$)	MW_{diff} (%)
$Cs(C_9H_{11})$	252	DSE	978	-74
$[Cs(C_9H_{11})]_4$	1008	DSE	978	+3
PMDETA (avg)	173	DSE	169	+6

6. References

(1) Gilman, H.; Pacevitz, H. A.; Baine, O. Benzylalkali compounds. *J. Am. Chem. Soc.* **1940**, *62*, 1514-1520.

(2) Morton, A. A.; Massengale, J. T.; Brown, M. L. The metalation of isopropylbenzene, condensations by sodium. *J. Am. Chem. Soc.* **1945**, *67*, 1620-1621.

(3) Gilman, H.; Tolman, L. Metalation of cumene by ethylpotassium. *J. Am. Chem. Soc.* **1946**, *68*, 522.

(4) Morton, A. A.; Little Jr, E. L. Polymerization X. Metalation of alkylaryl hydrocarbons and their use in the polymerization of butadiene. *J. Am. Chem. Soc.* **1949**, *71*, 487-489.

(5) Bryce-Smith, D. Organometallic compounds of the alkali metals. Part III. Metallation of alkylbenzenes by alkyl-sodium and -potassium compounds. The character of aromatic metallation reactions. *J. Chem. Soc.* **1954**, 1079-1088.

(6) Benkeser, R. A.; Hooz, J.; Liston, T. V.; Trevillyan, A. E. Factors governing orientation in metalation reactions. II. The metlation of isopropylbenzene with *n*-amylsodium and *n*-amylpotassium. *J. Am. Chem. Soc.* **1963**, *85*, 3984-3989.

(7) Broaddus, C. D. Homogeneous metalation of alkylbenzenes. *J. Org. Chem.* **1970**, *35*, 10-15.

(8) Crimmins, T. F.; Chan, C. M. Metalation of cumene with *n*-pentylsodium in the presence of *N*,*N*,*N'*,*N'*-tetramethylethylenediamine. Preparation of α-cumylsodium. *J. Org. Chem.* **1976**, *41*, 1870-1872.

(9) Xue, T. J.; Jones, M. S.; Ebdon, J. R.; Wilkie, C. A. Lithiation-alkylation of polystyrene occurs only on the ring. *J. Polym. Sci. Part A, Polym. Chem.* **1997**, *35*, 509-513.

(10) Shabanov, A. L.; Seidov, N. M.; Gasanova, U. A.; Kakhramanova, Z. O.; Gasanova, M. M. Metalation of toluene and cumene with alkali metal-crown ether complexes. *Russ. J. Org. Chem.* **2009**, *45*, 26-29.

(11) Anderson, D. E.; Malaspina, L. A.; Grabowsky, S.; Hevia, E. Synthesis and structure of neopentyl sodium: a hydrocarbon soluble reagent for controllled sodiation of non-activated substrates. *Angew. Chem. Int. Ed.* **2025**, *64*, e202511492.

(12) Gaussian 16, Revision C.01, Frisch, M. J.; Trucks, G. W.; Schlegel, H. B.; Scuseria, G. E.; Robb, M. A.; Cheeseman, J. R.; Scalmani, G.; Barone, V.; Petersson, G. A.; Nakatsuji, H.; Li, X.; Caricato, M.; Marenich, A. V.; Bloino, J.; Janesko, B. G.; Gomperts, R.; Mennucci, B.; Hratchian, H. P.; Ortiz, J. V.; Izmaylov, A. F.; Sonnenberg, J. L.; Williams-Young, D.; Ding, F.; Lipparini, F.; Egidi, F.; Goings, J.; Peng, B.; Petrone, A.; Henderson, T.; Ranasinghe, D.; Zakrzewski, V. G.; Gao, J.; Rega, N.; Zheng, G.; Liang, W.; Hada, M.; Ehara, M.; Toyota, K.; Fukuda, R.; Hasegawa, J.; Ishida, M.; Nakajima, T.; Honda, Y.; Kitao, O.; Nakai, H.; Vreven, T.; Throssell, K.; Montgomery, J. A., Jr.; Peralta, J. E.; Ogliaro, F.; Bearpark, M. J.; Heyd, J. J.; Brothers, E. N.; Kudin, K. N.; Staroverov, V. N.; Keith, T. A.; Kobayashi, R.; Normand, J.; Raghavachari, K.; Rendell, A. P.; Burant, J. C.; Iyengar, S. S.; Tomasi, J.; Cossi, M.; Millam, J. M.; Klene, M.; Adamo, C.; Cammi, R.; Ochterski, J. W.; Martin, R. L.; Morokuma, K.; Farkas, O.; Foresman, J. B.; Fox, D. J. Gaussian, Inc., Wallingford CT, 2016.

(13) Perdew, J. P.; Density-functional approximation for the correlation energy of the inhomogeneous electron gas. *Phys. Rev. B* **1986**, *33*, 8822.

(14) Becke, A. D.; Density-functional exchange-energy approximation with correct asymptotic behavior. *Phys. Rev. A* **1988**, *38*, 3098.

(15) Weigend, F.; Accurate Coulomb-fitting basis sets for H to Rn. *Phys. Chem. Chem. Phys.* **2006**, *8*, 1057.

(16) Grimme, S.; Antony, J.; Ehrlich, S.; Krieg, H.; A consistent and accurate ab initio parametrization of density functional dispersion correction (DFT-D) for the 94 elements H-Pu. *J. Chem. Phys.* **2010**, *132*,154104.

(17) Grimme, S.; Ehrlich, S.; Goerigk, L.; Effect of the damping function in dispersion corrected density functional theory. *J. Comput. Chem.* **2011**, *32*,1456

(18) AIMAll (Version 19.10.12), T. A. Keith, TK Gristmill Software, Overland Park KS, USA, 2019

(19) Contreras-García, J.; Johnson, E.R.; Keinan, S.; Chaudret, R.; Piquemal, J.-P.; Beratan, D. N.; Yang, W.; NCIPLOT: A Program for Plotting Noncovalent Interaction Regions. *J. Chem. Theory Comput.* **2011**, *7*, 625.
(20) Rigaku OD **2021**. *CrysalisPRO*. Rigaku Ltd. Yarnton, Oxfordshire, England.
(21) Sheldrick, G. M.; SHELXT – Integrated space-group and crystal-structure determination. *Acta Cryst. A*. **2015**, *71*, 3-8.
(22) Sheldrick, G. M; Crystal structure refinement with SHELXL. *Acta Cryst. C* **2015**, *71*, 3-8.
(23) Bourhis, L. J.; Dolomanov, O. V.; Gildea, R. J.; Howard, J. A. K.; Puschmann, H.; OLEX2: a complete structure solution, refinement and analysis program. *J. Appl. Crystallogr.* **2009**, *42*, 339-341.
(24) Farrugia, L. J.; WinGX and ORTEP for Windows: an update. *J. Appl. Cryst.* **2012**, *45*, 849-854.
(25) Bachmann, S.; Gernert, B.; Stalke, D.; Solution structures of alkali metal cyclopentadienides in THF estimated by ECC-DOSY NMR-spectroscopy (incl. software). *Chem. Commun.* **2016**, *52*, 12861-12864;
(26) Bachmann, S.; Neufeld, R.; Dzemski, M.; Stalke, D.; New External Calibration Curves (ECCs) for the Estimation of Molecular Weights in Various Common NMR Solvents. *Chem. Eur. J.* **2016**, *22*, 8462-8465.
(27) Neufeld, R.; Stalke, D.; Accurate molecular weight determination of small molecules via DOSY-NMR by using external calibration curves with normalized diffusion coefficients. *Chem. Sci.* **2015**, *6*, 3354-3364.
